# Spatio-temporal disease risk estimation using clustering-based adjacency modelling

**DOI:** 10.1177/09622802221084131

**Published:** 2022-03-14

**Authors:** Xueqing Yin, Gary Napier, Craig Anderson, Duncan Lee

**Affiliations:** 1 School of Mathematics and Statistics, 3526 University of Glasgow, UK

**Keywords:** Clustering, conditional autoregressive models, neighbourhood matrix, spatio-temporal modelling

## Abstract

Conditional autoregressive models are typically used to capture the spatial autocorrelation present in areal unit disease count data when estimating the spatial pattern in disease risk. This correlation is represented by a binary neighbourhood matrix based on a border sharing specification, which enforces spatial correlation between geographically neighbouring areas. However, enforcing such correlation will mask any discontinuities in the disease risk surface, thus impeding the detection of clusters of areas that exhibit higher or lower risks compared to their neighbours. Here we propose novel methodology to account for these clusters and discontinuities in disease risk via a two-stage modelling approach, which either forces the clusters/discontinuities to be the same for all time periods or allows them to evolve dynamically over time. Stage one constructs a set of candidate neighbourhood matrices to represent a range of possible cluster/discontinuity structures in the data, and stage two estimates an appropriate structure(s) by treating the neighbourhood matrix as an additional parameter to estimate within a Bayesian spatio-temporal disease mapping model. The effectiveness of our novel methodology is evidenced by simulation, before being applied to a new study of respiratory disease risk in Greater Glasgow, Scotland from 2011 to 2017.

## 1 Introduction

Disease risk varies in space and time, and is affected by risk factors such as environmental exposures (e.g. air pollution) and population behaviours (e.g. smoking). Health agencies routinely produce disease maps to identify high-risk areas and highlight health inequalities,^1^ by displaying raw disease rates for n non-overlapping areal units that comprise the study region. However, raw disease rates can exhibit extreme values for rare diseases or for areas with small populations, and also ignore the potential spatial autocorrelation between nearby areas, which collectively leads to poor estimation of disease risk. Additionally, visually examining maps of raw disease rates does not allow the systematic identification of clusters of areas that exhibit higher risks compared to their geographical neighbours, which health agencies will want to identify for risk reduction strategies.

Therefore, Bayesian hierarchical models have been widely developed for modelling areal unit disease count data, which allow for their spatially correlated nature by incorporating a set of random effects that are assigned a conditional autoregressive (CAR) prior distribution (see Besag et al.^2^ and Leroux et al.^3^). This class of models has been extended to the spatio-temporal domain to allow the estimation of spatio-temporal trends, including by Bernardinelli et al.,^4^ Knorr-Held,^5^ Ugarte et al.,^6^ Rushworth et al.^7^ and Napier et al.^8^ In all of these models spatial autocorrelation is induced by an n×n neighbourhood matrix $W=(w_{ij})$ , where typically $w_{ij}=1$ if areal units (i,j) are spatially close (e.g. share a common border) and $w_{ij}=0$ otherwise. Using this specification the estimation of disease risk borrows strength between geographically adjacent areal units, thus assuming the risk surface is globally spatially smooth. However in practice, some pairs of neighbouring areas are likely to exhibit very different disease risks, which can be driven by social, economic or environmental characteristics of adjacent neighbourhoods.^9^ Thus using the border sharing specification defined above is likely to be inappropriate for capturing the complex spatial correlation structures observed in areal unit disease count data. Furthermore, few existing studies assess the appropriateness of W for the data they model, nor do they assess the sensitivity of their results to changing its specification. This is in sharp contrast to the related field of geostatistics, where variogram analysis is routinely used to identify an appropriate spatial autocorrelation structure for the data.

A number of existing approaches have allowed for spatial discontinuities in the disease risk surface, by identifying clusters of areas that exhibit elevated or reduced risks compared to their neighbours. One of the earliest approaches is scan statistics,^10^ which can be implemented with the SaTScan software. However, scan statistics aim to identify a relatively small number of clusters of areas exhibiting high-risks, rather than partitioning the risk surface into different risk levels or estimating disease risk across the entire study region. In contrast, Knorr-Held and Raßer^11^ identify clusters using a piecewise constant risk model that is implemented using a reversible jump Markov chain Monte Carlo (MCMC) algorithm, but it involves a computationally expensive and complex algorithm for which publicly available code is not available. Similarly, Wakefield and Kim^12^ and Anderson et al.^13^ capture risk discontinuities by utilising a piecewise constant cluster model. The latter is a two-stage approach, which first obtains a set of candidate cluster configurations, and then fits separate Bayesian hierarchical models to each configuration and chooses the best one using a model selection criterion. Adin et al.^14^ extends Anderson et al.^13^ by replacing the cluster fixed effects with random effects, and considering a spatio-temporal rather than a spatial setting. However, both papers treat the identification of the best cluster configuration as a model comparison problem, and thus have the common computational limitation of fitting multiple Bayesian models separately, as well as ignoring the uncertainty about the number of clusters in the data.

Anderson et al.^15^ address these deficiencies in a purely spatial setting, by estimating disease risk and the cluster structure simultaneously in a single model, thus quantifying the uncertainty in the estimated cluster structure. They do this by a two-stage approach, which first uses agglomerative hierarchical clustering to create a set of candidate cluster structures, each of which corresponds to a candidate neighbourhood matrix. Then in stage two they fit a single model that treats W as a parameter to be estimated, and assign it a discrete uniform prior whose values are the set of candidate neighbourhood matrices previously constructed. This paper develops a novel two-stage approach for simultaneously identifying clusters/discontinuities and estimating the spatio-temporal trend in disease risk, which extends the approach of Anderson et al.^15^ in two main ways. First, we consider a spatio-temporal rather than a purely spatial setting, and propose approaches that either force the spatial clusters/discontinuities to be the same for all time periods or allow them to evolve dynamically over time. Second, we construct a much bigger set of candidate cluster/discontinuity structures using a range of clustering methods, which gives much greater flexibility in cluster identification compared to Anderson et al.^15^ who just used agglomerative hierarchical clustering.

This methodology is motivated by a new study of respiratory disease risk in the Greater Glasgow and Clyde Health Board during the time period from 2011 to 2017, and our main aim is to identify the, possibly temporally evolving, cluster/discontinuity structures in disease risk. The remainder of this paper is organised as follows. Section 2 presents the motivating data set and our questions of interest, as well as giving a brief literature review of spatio-temporal disease risk models. Section 3 presents our new methodology, while the efficacy of this approach is evidenced using simulation in Section 4. Then Section 5 applies the methodology to the motivating application, while Section 6 finishes the paper with a concluding discussion.

## 2 Background

### 2.1 Motivating study

**Figure 1.**
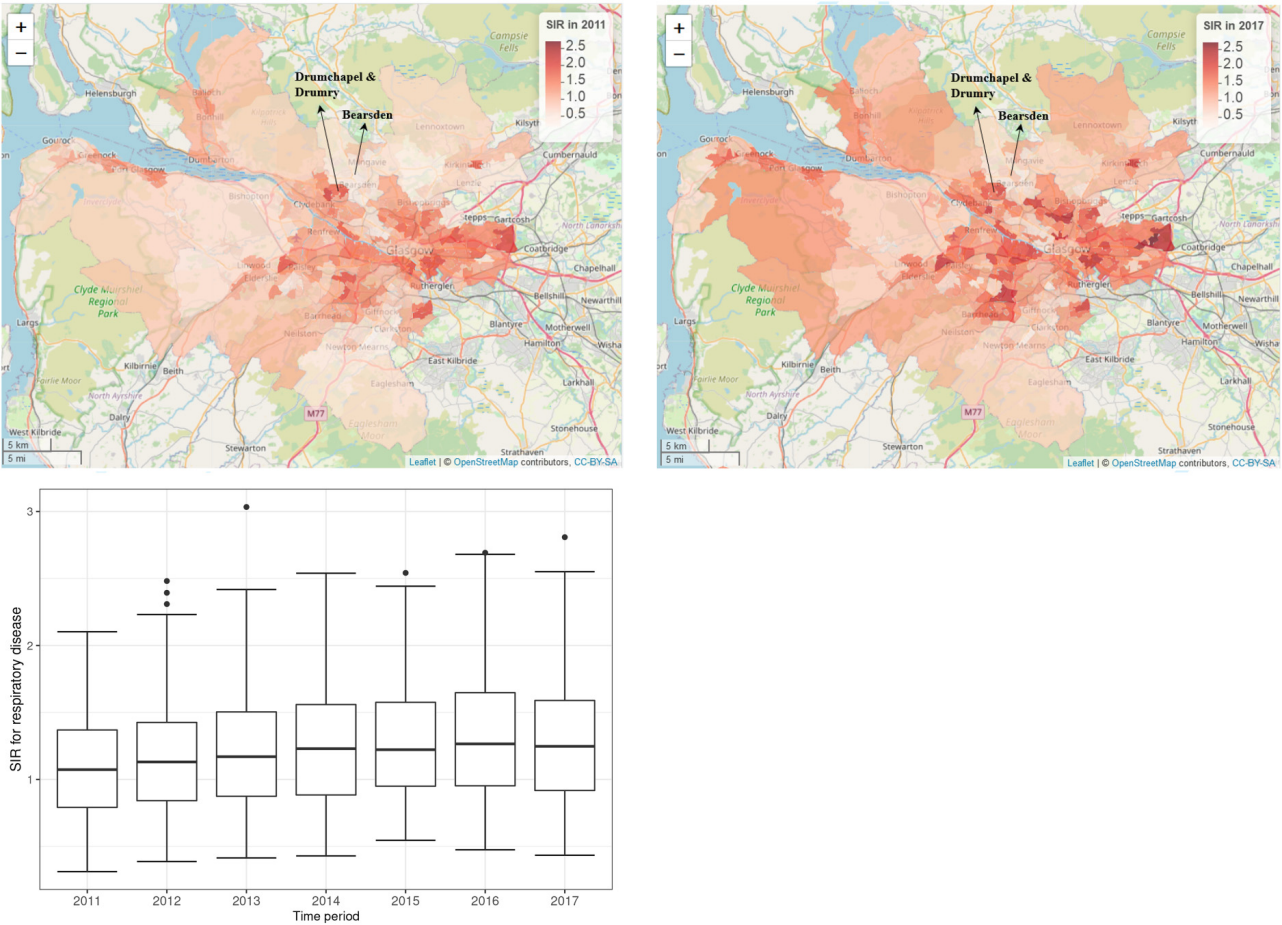
Maps of the standardised incidence ratio (SIR) for respiratory disease in the intermediate zones in the Greater Glasgow and Clyde Health Board in 2011 (the top-left panel) and 2017 (the top-right panel). The bottom panel shows boxplots of the SIR over time.

The methodology proposed in Section 3 is motivated by a study of respiratory disease (defined using the International Classification of Disease tenth revision by codes J00-J99) in the Greater Glasgow and Clyde Health Board region in Scotland between 2011 and 2017. The study region, as shown in Figure 1, contains the city of Glasgow and the surrounding rural areas, and is split in two by the River Clyde running north west through the region. The study region has a population of around 1,000,000 people and is split up into n=257 intermediate zones (IZs), each with an average population of approximately 4000 residents. The disease data, $Y=\{Y_{it}\}$ , available from Public Health Scotland, are the yearly counts of the numbers of hospital admissions with a primary diagnosis of respiratory disease for i=1,…,n(=257) IZs for t=1,…,T(=7) years. Additionally, the expected number of respiratory hospitalisations is calculated for each year and IZ using indirect standardisation to adjust for different population sizes and age and sex structures across the IZs, and are denoted here by $E=\{E_{it}\}$ . These expected counts are based on Scotland-wide age-sex specific respiratory hospitalisation rates, because it allows us to examine how the risk of disease in Glasgow compares to the national average, which is the benchmark often used by Public Health Scotland when examining the spatial patterns in disease risk.

The standardised incidence ratio (SIR) is an exploratory estimate of disease risk calculated by ${\text{SIR}}_{it}\;=\frac{Y_{it}}{E_{it}}$ , where a value greater than 1 corresponds to an increased level of risk compared to the Scottish average while a value less than 1 indicates a decreased level of risk. For instance, an SIR of 1.2 corresponds to a risk 20% higher than the Scottish average during the study period. The spatial patterns in the SIR for 2011 (the first year of data) and 2017 (the last year of data) are displayed in the top panels of Figure 1. They show that higher respiratory disease risks are mainly in the East End of Glasgow (the east of the map) and along the southern bank of the River Clyde, which includes the socio-economically deprived areas of Easterhouse and Govan respectively, both of which are well known to suffer from multi-generational poverty.^16^ In addition, there are numerous pairs of neighbouring areas where a discontinuity in disease risk appears to exist, suggesting the presence of clusters of areas that exhibit elevated risks compared with their neighbours. For example, in 2017 Drumchapel and Drumry to the north west of the city exhibits a vastly higher SIR value (SIR = 1.85) than its neighbour Bearsden (SIR = 0.53). Therefore, the common approach in the literature of assuming that all pairs of neighbouring areal units exhibit similar disease risks is clearly not appropriate, which motivates the spatio-temporal clustering model proposed below.

The spatial patterns in the SIR are fairly similar for each year, with an average Pearson’s correlation coefficient of 0.844 between each pair of years. This suggests that while any clusters identified in disease risk may evolve slightly over time, one would not expect a large change in the clusters from year to year. This gradual change is likely to be because respiratory related hospitalisations are a marker of chronic rather than epidemic disease, and hence any change would likely be gradual and due to factors such as the gentrification of an area. The temporal trend in the SIR is shown in the bottom panel of Figure 1, which shows that overall there has been a slight increase in the SIR over the 7-year period, with a mean value of 1.10 in 2011 compared to 1.28 in 2017. There also appears to be increased spatial variation in risk in the later years, with standard deviations over space of 0.39 in 2011 and 0.46 in 2017. Finally, we do not collect any covariate data for this study, because our aim is to estimate the spatio-temporal trend in disease risk and its spatial cluster structure, rather than the drivers of elevated disease risks. Furthermore, as the clusters identified by our methodology are in the random effects surface, then including covariates in the model would mean that the clustering/discontinuities relate to the residual risk surface after covariate adjustment. In contrast, by not including covariates in the model the random effects and risk surfaces have the same spatial structure (see Section 3), and thus any clusters/discontinuities identified also relate to disease risk.

### 2.2 Review of spatio-temporal modelling of areal unit count data

#### 2.2.1 Notation and data likelihood

Consider a study region partitioned into n non-overlapping areal units indexed by i∈{1,…,n} , where data are collected for t∈{1,…,T} consecutive time periods. As previously described $Y=\{Y_{it}\}$ and $E=\{E_{it}\}$ , respectively, denote the set of observed and expected disease counts, while a vector of covariates (if needed) is given by $x_{it}$ for area i and time period t . The model we outline in this paper is the most general form that includes covariates, but as previously described the application in this paper only includes an intercept term in the model. As the response variable is a count the data likelihood model commonly used is given by $Y_{it}|E_{it},R_{it}∼\text{Poisson}(E_{it}R_{it})$ , where $R_{it}$ represents disease risk in areal unit i during time period t and is on the same scale as the SIR.

#### 2.2.2 Spatio-temporal risk model

The spatio-temporal structure in risk $\left\{R_{it}\right\}$ is typically modelled by both covariates and random effects, and a large number of different random effects structures have been proposed. An appropriate choice of structure depends on both the aims of the analysis and the trends observed in the data, and the first paper in this area proposed using spatially correlated linear time trends^4^ for each area. Probably the most widely used structure to date was proposed by Knorr-Held,^5^ which decomposes disease risk into separate spatial and temporal main effects and an additional spatio-temporal interaction term. More recently, two popular spatio-temporal structures were proposed by Rushworth et al.^7^ and Napier et al.,^8^ respectively. Rushworth et al.^7^ modelled the risk surface as a spatially autocorrelated multivariate first-order autoregressive process, while Napier et al.^8^ built on the model of Waller et al.^17^ by using a region-wide temporal trend and separate spatial processes for each year. In this paper, we use the latter of these, because it is the only one of the aforementioned approaches to have a separate spatial process with a potentially different neighbourhood matrix W for each time period. Having a separate W for each time period is crucial for the methodology proposed in the next section, because it is the mechanism by which we estimate the temporally evolving clusters/discontinuities in disease risk. The risk model proposed by Napier et al.^8^ that our approach is based on is given by the Poisson log-linear specification

(1) $$\begin{array}{rl} Y_{it}|E_{it},R_{it} & ∼\text{Poisson}(E_{it}R_{it}),\;\;i=1,\ldots ,n;\;\;t=1,\ldots ,T\\ \ln\;(R_{it}) & =x_{it}^{⊤}\beta +\phi_{it}+\theta_{t} \end{array}$$

where β is a vector of regression parameters. The residual spatio-temporal structure (after covariate adjustment) is modelled by an overall temporal trend $\theta =(\theta_{1},\ldots ,\theta_{T})$ and a separate spatial surface at each time period t , $\phi_{t}=(\phi_{1t},\ldots ,\phi_{nt})$ . Each spatial surface $\phi_{t}$ is modelled by the Leroux CAR prior,^3^ which induces spatial autocorrelation into the random effects via a neighbourhood matrix W that determines which pairs of areal units are close together. Here we adopt the commonly used *sharing a common border specification*, where $w_{ij}=1$ if areal units (i,j) share a common geographical border, and $w_{ij}=0$ otherwise. Based on this matrix the Leroux CAR prior^3^ for $\phi_{t}$ is specified by n univariate full conditional distributions, which for area i is given by

(2) $$\phi_{it}|\phi_{-i,t},W∼\text{N}\left(\frac{\rho_{s}\sum_{\;j=1}^{n}w_{ij}\phi_{\;jt}}{\rho_{s}\sum_{\;j=1}^{n}w_{ij}+1-\rho_{s}},\frac{\tau_{t}^{2}}{\rho_{s}\sum_{\;j=1}^{n}w_{ij}+1-\rho_{s}}\right)$$

where $\phi_{-i,t}=(\phi_{1,t},\ldots ,\phi_{i-1,t},\phi_{i+1,t},\ldots ,\phi_{n,t})$ . The strength of the spatial autocorrelation is controlled by a temporally invariant parameter $\rho_{s}$ , where a value of 1 indicates strong dependence in space (corresponding to the intrinsic CAR model^2^) and a value of 0 indicates independence (as $\phi_{it}∼\text{N}(0,\tau_{t}^{2})$ ). Additionally, $\tau_{t}^{2}$ is a temporally-varying variance parameter, thus allowing the amount of spatial variation in the data to change over time. The joint multivariate Gaussian distribution for $\phi_{t}$ corresponding to the above is $\phi_{t}∼\text{N}(0,\tau_{t}^{2}Q(\rho_{s},W{)}^{-1})$ , where $Q(\rho_{s},W)=\rho_{s}(\text{d}iag(W1)-W)+(1-\rho_{s})I$ , 1 is an n×1 vector of ones and I is an n×n identity matrix. The partial correlation between $\left(\phi_{it},\phi_{\;jt}\right)$ conditioning on the remaining spatial random effects (denoted $\phi_{-ijt}$ ) specified by this model is

(3) $$\text{Corr}\left(\phi_{it},\phi_{\;jt}|\phi_{-ijt}\right)=\frac{\rho_{s}w_{ij}}{\sqrt{\left(\rho_{s}\sum_{v=1}^{n}w_{iv}+1-\rho_{s}\right)\left(\rho_{s}\sum_{v=1}^{n}w_{\;jv}+1-\rho_{s}\right)}}$$

Equation (3) shows that $\left(\phi_{it},\phi_{\;jt}\right)$ are modelled as partially correlated if $w_{ij}=1$ , otherwise, the partial correlation between $\left(\phi_{it},\phi_{\;jt}\right)$ is 0 and they are modelled as conditionally independent. Hence the neighbourhood matrix W determines the spatial autocorrelation structure imposed by the model. Thus using the border sharing rule the spatial random effect $\phi_{it}$ is forced to be correlated with its geographical neighbours if $\rho_{s}$ is estimated as close to one, meaning that any discontinuities in the spatial surface are smoothed over in the estimation. This not only leads to poorer risk estimation in the presence of discontinuities as we show in Section 4, but also does not allow a mechanism for identifying these discontinuities that correspond to cluster boundaries. Finally, Napier et al.^8^ modelled the temporal trend $\theta =(\theta_{1},\ldots ,\theta_{T})$ by a one dimensional Leroux CAR prior, and further details can be found in their paper.

#### 2.2.3 Inference

Inference in these spatio-temporal areal unit models is typically based in a Bayesian setting, using either MCMC simulation or integrated nested Laplace approximations (INLA). We adopt the former approach here, because we treat W as a random parameter to be estimated which is not straightforward to do using INLA.

## 3 Methodology

We propose a novel two-stage modelling approach that jointly estimates the spatio-temporal pattern in disease risk and identifies clusters of areas with elevated or reduced risks compared to their geographical neighbours. In stage one a range of clustering methods are used to identify a large collection of plausible candidate cluster structures for the data, each of which is then used to create a candidate neighbourhood matrix. In stage two, a Bayesian hierarchical model is proposed for the data, which jointly estimates the spatio-temporal pattern in disease risk and the most appropriate neighbourhood matrix corresponding to a given cluster structure. We propose two different variants of the model, with variant A having spatial clusters that remain fixed during the entire study period, while in variant B the clusters vary dynamically over time.

### 3.1 Stage 1 – Generating neighbourhood matrices representing clusters/discontinuities

A collection of candidate cluster structures for disease risk are estimated based on c=1,…,M different clustering methods, and the M=8 methods considered here are summarised in Table 1. The methods include k-means^18^ clustering, k-medoids^19^ clustering, hierarchical agglomerative^20^ clustering with centroid, complete, average and Ward linkage, divisive^21^ clustering and expectation–maximisation^22^ clustering. Note, agglomerative clustering with single linkage is not considered due to its limitation of frequently suffering from the chaining effect.^23^ These clustering methods are applied to the data without regard to the spatial positions of the areal units, because the spatial correlation in the data is modelled by random effects as described above. Thus the clusters identified represent the number of different risk levels rather than the number of spatially distinct clusters, meaning that a single *‘cluster’* will likely contain groups of areas that are not spatially connected by W .

**Table 1.** A key for each value of c and its associated clustering method.

c	**clustering method**
c=1	k-means clustering (kmeans)
c=2	k-medoids clustering (kmedoids)
c=3	Hierarchical agglomerative clustering with centroid linkage (agg_centroid)
c=4	Hierarchical agglomerative clustering with complete linkage (agg_complete)
c=5	Hierarchical agglomerative clustering with average linkage (agg_average)
c=6	Hierarchical agglomerative clustering with ward linkage (agg_ward)
c=7	Hierarchical divisive clustering (div)
c=8	Expectation–maximisation clustering (EM)

Each clustering method c is used to compute k=1,…,K distinct cluster structures, where structure k contains k clusters. Note, these K cluster structures are not necessarily nested, as for example a k-means solution with four clusters is not obtained by splitting one of the clusters from the k-means with three solutions into two. The value K is chosen to be an upper limit for the number of clusters one would expect to find in the data, which must be specified by the user. Here we set K=10 as a conservative overly large choice, because as described above this represents the number of distinct risk levels and not the number of spatially contiguous clusters. These candidate cluster structures are incorporated into the disease risk model by specifying a set of candidate neighbourhood matrices, which means they relate to the random effects surface $\left\{\phi_{it}\right\}$ . Therefore we estimate $\left\{\phi_{it}\right\}$ from the data and the general model (1) by

(4) $${\tilde{\phi }}_{it}=\ln\;\left(\frac{E(Y_{it})}{E_{it}}\right)-x_{it}^{⊤}\beta -\theta_{t}\approx \ln\;\left(\frac{Y_{it}}{E_{it}}\right)-x_{it}^{⊤}\hat{\beta }$$

This approximation replaces the unknown $E(Y_{it})$ with the observed data $Y_{it}$ , and the temporal random effects are removed as they do not vary over space and hence do not affect the spatial cluster structure. Finally, the regression parameters are estimated for this initial stage assuming independence via maximum likelihood estimation, and are denoted above by $\hat{\beta }$ . These estimated spatial random effects $\left\{{\tilde{\phi }}_{it}\right\}$ are used to construct candidate cluster structures and corresponding neighbourhood matrices as described below for variants A (static) and B (dynamic) of our model.

#### 3.1.1 Variant A: Constant cluster structure over time

Temporally constant clusters are obtained by applying each clustering method to $\tilde{\phi }=({\tilde{\phi }}_{1},\ldots ,{\tilde{\phi }}_{n})$ , where ${\tilde{\phi }}_{i}=({\tilde{\phi }}_{i1},\ldots ,{\tilde{\phi }}_{iT})$ . The cluster structure obtained from method c with k clusters is used to create a candidate neighbourhood matrix $W^{\left(c,k\right)}$ as follows:

(5) $$w_{ij}^{\left(c,k\right)}=\left\{ \begin{array}{c} 1,\;\;\mathrm{if}\;\mathrm{areal}\;\mathrm{units}\;(i,j)\;\mathrm{share}\;a\;\mathrm{common}\;\mathrm{border}\;\mathrm{and}\;\mathrm{are}\;\mathrm{in}\;\mathrm{the}\;\mathrm{same}\;\mathrm{cluster},\\ 0,\;\;\mathrm{otherwise}. \end{array}\right.$$

Thus there is a one-to-one relationship between a candidate cluster structure and its corresponding neighbourhood matrix, and as trivially the border sharing matrix equals all of $\left\{W^{\left(1,1\right)},\ldots ,W^{\left(M,1\right)}\right\}$ this leads to (K−1)×M+1 candidate cluster structures in total. Altering the border sharing neighbourhood matrix in this way to allow for clusters means that if areas (i,j) share a border and are in the same cluster (i.e. have similar data values) then their random effects will be modelled as partially correlated (see equation (3)). In contrast, if they share a border but are in different clusters (i.e. have very different data values) then their random effects will be modelled as conditionally independent, thus not enforcing spatial smoothing between them.

#### 3.1.2 Variant B: Temporally varying cluster structures

In variant A, the spatial clusters do not change over time, which allows one to estimate an overall average cluster structure in the data across the entire study. However this may not be realistic in practice, because different areas can have different temporal trends in disease risk leading to evolution in the spatial cluster structure over time. To account for this we adjust the clustering method in variant A by estimating a separate spatial cluster structure for each time period. This is achieved by applying clustering method c with k clusters to ${\tilde{\phi }}_{t}=({\tilde{\phi }}_{1t},\ldots ,{\tilde{\phi }}_{nt})$ separately for each time period t , yielding a candidate neighbourhood matrix $W^{\left(c,k,t\right)}$ defined as follows:

(6) $$w_{ij}^{\left(c,k,t\right)}=\left\{ \begin{array}{c} 1,\;\;\mathrm{if}\;\mathrm{areal}\;\mathrm{units}\;(i,j)\;\mathrm{share}\;a\;\mathrm{common}\;\mathrm{border}\;\mathrm{and}\;\mathrm{are}\;\mathrm{in}\;\mathrm{the}\;\mathrm{same}\;\mathrm{cluster}\;\mathrm{during}\;\mathrm{time}\;\mathrm{period}\;t,\\ 0,\;\;\mathrm{otherwise}. \end{array}\right.$$

This algorithm leads to (K−1)×M×T+1 candidate cluster structures in total, including (K−1)×M for each time period t and the additional border sharing specification.

### 3.2 Stage 2 – Bayesian spatio-temporal modelling

The second stage of our method fits a model to the data that jointly estimates the spatio-temporal trend in disease risk and an appropriate cluster/discontinuity structure(s), the latter being achieved by treating W as a parameter to be estimated from the set of candidates generated in stage 1. The model we propose is based on Napier et al.^8^ described in Section 2.2, because its separate random effects surfaces for each time period allow for different neighbourhood matrices to be specified in each case which is necessary under variant B of the model. The first level of the model is given by

(7) $$\begin{array}{rl} Y_{it}|E_{it},R_{it} & ∼\text{Poisson}(E_{it}R_{it})\;\;i=1,\ldots ,n;\;\;t=1,\ldots ,T\\ \ln\;(R_{it}) & =x_{it}^{⊤}\beta +\phi_{it}+\theta_{t}\\ \beta_{j} & ∼\text{N}(0,1000),\text{ for }\;j=0,\ldots ,p \end{array}$$

The residual spatio-temporal variation in the data is decomposed into an overall temporal trend common to all areal units and separate spatial surfaces for each time period. The former is denoted by $\theta =(\theta_{1},\ldots ,\theta_{T})$ and modelled by the first-order autoregressive process

(8) $$\begin{array}{rl} \theta_{t}|\theta_{t-1} & ∼\text{N}\left(\alpha \theta_{t-1},\sigma^{2}\right)\text{ for }\;t=2,\ldots ,T\\ \theta_{1} & ∼\text{N}\left(0,\sigma^{2}\right)\\ \alpha & ∼\text{Uniform}(0,1)\\ \sigma^{2} & ∼\text{Inverse-Gamma}(1,0.01) \end{array}$$

Here α∈[0,1] is the temporal autoregressive parameter, with α=1 indicating strong temporal dependence (a first-order random walk), while α=0 corresponds to independence across time. We assign a uniform prior on the interval [0,1] to α , while a conjugate Inverse-Gamma prior is assigned to the process variance $\sigma^{2}$ . The spatial surface at time period t is captured by $\phi_{t}=(\phi_{1t},\ldots ,\phi_{nt})$ , which is modelled by a separate Leroux CAR prior^3^ for each time period. We allow for different spatial variances for each time period because the exploratory analysis in Section 2.1 suggested that the level of spatial variation may change over time. For both model variants A and B, the neighbourhood matrix is treated as a parameter to be estimated, and the model specifications are given below.

#### 3.2.1 Variant A: Constant cluster structure over time

In this model variant, there is a single neighbourhood matrix $\tilde{W}$ that is common to all time periods, and the spatial random effects $\phi_{t}$ are modelled by

$$\begin{array}{rl} \phi_{t} & ∼\text{N}\left(0,\tau_{t}^{2}Q(\rho_{s},\tilde{W}{)}^{-1}\right)\\ \tilde{W} & ∼\text{Discrete \textbackslash{};uniform}\left(W^{\left(1,1\right)},W^{\left(1,2\right)},\ldots ,W^{\left(1,K\right)},W^{\left(2,2\right)},\ldots ,W^{\left(2,K\right)},\ldots ,W^{\left(M,2\right)},\ldots ,W^{\left(M,K\right)}\right) \end{array}$$

Here $Q(\rho_{s},\tilde{W})$ is the spatial precision matrix corresponding to the Leroux CAR prior, which was defined in the previous section. This matrix depends on the neighbourhood matrix $\tilde{W}$ , which is assigned a discrete uniform prior whose possible values are the set of candidates corresponding to the cluster structures estimated in stage 1. Finally, the variance and spatial dependence parameters are assigned weakly informative Inverse-Gamma ( $\tau_{t}^{2}∼\text{Inverse-Gamma}(1,0.01)$ ) and uniform ( $\rho_{s}∼\text{Uniform}(0,1)$ ) priors, respectively.

#### 3.2.2 Variant B: Temporally varying cluster structures

In this model variant, there is a different neighbourhood matrix ${\tilde{W}}_{t}$ for each time period t , and the spatial random effects $\phi_{t}$ are modelled by

$$\begin{array}{rl} \phi_{t} & ∼\text{N}\left(0,\tau_{t}^{2}Q(\rho_{s_{t}},{\tilde{W}}_{t}{)}^{-1}\right)\\ {\tilde{W}}_{t} & ∼\text{Discrete \textbackslash{};uniform}\left(W^{\left(1,1,t\right)},W^{\left(1,2,t\right)},\ldots ,W^{\left(1,K,t\right)},W^{\left(2,2,t\right)},\ldots ,W^{\left(2,K,t\right)},\ldots ,W^{\left(M,2,t\right)},\ldots ,W^{\left(M,K,t\right)}\right) \end{array}$$

Here $Q(\rho_{s_{t}},{\tilde{W}}_{t})$ again corresponds to the Leroux CAR prior, where in this model variant the spatial dependence parameter $\rho_{s_{t}}$ changes over time as the neighbourhood matrix also varies over time. As before the set of candidate neighbourhood matrices at time t that make up the discrete uniform prior for ${\tilde{W}}_{t}$ are obtained from the candidate cluster structures generated in stage 1. In common with variant A, the model specification is completed with $\tau_{t}^{2}∼\text{Inverse-Gamma}(1,0.01)$ and $\rho_{s_{t}}∼\text{Uniform}(0,1)$ .

For both model variants, the clustering stage elicits multiple candidate neighbourhood matrices that are equal to the traditional border sharing W , which occur when the number of clusters k=1 for each clustering method. Therefore only one of these is included in the discrete uniform prior to avoid the border sharing W being given a larger prior weight compared to the other candidate values. We include this border sharing W in the model because it corresponds to a globally spatially smooth risk surface with no clusters. Additionally, to achieve identifiability, all sets of spatial and temporal random effects are zero-mean centred.

Here $\left(\rho_{s},\rho_{s_{t}}\right)$ control the level of spatial autocorrelation globally across the study region, with values close to 1 corresponding to strong spatial autocorrelation while a value of zero corresponds to spatial independence. However, in our two model variants, the spatial autocorrelation structure is modelled locally for each pair of neighbouring areas by estimating an appropriate neighbourhood matrix for the data, which may make the estimation of a single global parameter redundant. Thus in the simulation study in Section 4, we compare the performance of both model variants when estimating $\left(\rho_{s},\rho_{s_{t}}\right)$ in the model and also when fixing them at $\rho_{s},\rho_{s_{t}}=0.99$ . The latter is chosen because it is close to one and hence enforces strong spatial autocorrelation globally, whose structure is then adjusted locally by estimating the neighbourhood matrix. Note, we do not set $\rho_{s},\rho_{s_{t}}=1$ because our model could produce a candidate neighbourhood matrix where an areal unit has no neighbours due to it being a singleton cluster. This will cause $\sum_{\;j=1}^{n}w_{ij}=0$ for the area i in question, which leads to an infinite mean and variance for $\phi_{it}$ from (2).

### 3.3 Inference

Inference is carried out in a Bayesian setting via MCMC simulation, using both the Metropolis-Hastings algorithm^24,25^ and Gibbs sampling.^26^ The only non-standard step is the updating of the neighbourhood matrices $\tilde{W}$ or ${\tilde{W}}_{t}$ , which is achieved by using a Metropolis-Hastings step consisting of two MCMC moves, which are outlined below. Note, the step is outlined for variant A of the model, and the updating step for variant B is analogous.

1. If the current value of $\tilde{W}$ in the Markov chain is $W^{\left(c,k\right)}$ , then a new value $W^{\left(c,l\right)}$ is proposed uniformly from the set of candidate matrices $\left(W^{\left(c,k-s\right)},\ldots ,W^{\left(c,k-1\right)},W^{\left(c,k+1\right)},\ldots ,W^{\left(c,k+s\right)}\right)$ , which is the candidate cluster structures generated from the same clustering method but with a different number of clusters. Here s is a parameter controlling the acceptance rates and mixing of the update, and exploratory model runs suggested that s=2 leads to good estimation performance.
2. If the current value of $\tilde{W}$ after the first move is $W^{\left(c,k^{{}^{\prime }}\right)}$ , then a new proposal $W^{\left(h,k^{{}^{\prime }}\right)}$ is drawn uniformly from the set $\left(W^{\left(1,k^{{}^{\prime }}\right)},\ldots ,W^{\left(c-1,k^{{}^{\prime }}\right)},W^{\left(c+1,k^{{}^{\prime }}\right)},\ldots ,W^{\left(M,k^{{}^{\prime }}\right)}\right)$ , which is the candidate cluster structures with the same number of clusters but generated from a different clustering method.

Since the neighbourhood matrix follows a discrete distribution, the posterior mode of $(\tilde{W}$ , ${\tilde{W}}_{t})$ , representing the most likely occurring cluster structure across all the MCMC samples, is used to estimate the optimal cluster/discontinuity structure. In contrast, the remaining parameters are summarised by their posterior medians. The MCMC algorithm for fitting the model was developed and implemented in R^27^ and C++ via the R package Rcpp,^28^ and is available from https://github.com/XueqingYin/ST-model.

## 4 Simulation study

In this section, we present a simulation study to comprehensively quantify the performance of our methodology. The study assesses the performance of both model variants (A – static and B – time varying), and in both cases, we compare models where the global spatial dependence parameters $\left(\rho_{s},\rho_{s_{t}}\right)$ are fixed at 0.99 or estimated within the model. Thus in the study, we compare five different models, where **ST-A** and **ST-B** denote model variants A and B, respectively, where $\rho_{s},\rho_{s_{t}}=0.99$ , while ST-A* and ST-B* denote model variants A and B, respectively, where $\left(\rho_{s},\rho_{s_{t}}\right)$ are estimated within the model. Finally, model **ST-N** is the existing non-cluster model proposed by Napier et al.^8^ (see Section 2.2). Our aims in this study are to illustrate the improved risk estimation delivered by our models compared to a similar non-clustering alternative, and also to quantify the accuracy of the resulting cluster identification.

### 4.1 Data generation

Simulated disease count data $\left\{Y_{it}\right\}$ are generated from the Poisson log-linear model (7) for the n=257 IZs that comprise the Greater Glasgow study region for T=7 time periods. The template for the expected disease counts $\left\{E_{it}\right\}$ is based on the motivating study data, whose values range between 12.61 and 160.15 in a single IZ and year with a median of 74.09. However, to explore the impact of disease prevalence on model performance, these $\left\{E_{it}\right\}$ values are divided by the scale factors (SFs) of 1, 2 and 4. Thus SF=1 corresponds to the study data, SF=2 corresponds to having a smaller number of expected counts, while SF=4 represents a rare disease that has very small expected counts.

Disease risks $\left\{R_{it}\right\}$ are generated by simulating both spatial $\left(\{\phi_{it}\}\right)$ and temporal $\left(\{\theta_{t}\}\right)$ random effects, and as previously described covariates are not included. The temporal random effects are generated from the Gaussian AR(1) model given by (8), where we fix α=0.9 and $\sigma^{2}=0.1$ . The spatially correlated random effects for each time period $\phi_{t}=(\phi_{1t},\ldots ,\phi_{nt})$ are generated from a multivariate Gaussian distribution with the spatially correlated precision matrix proposed by Leroux et al.,^3^ where we fix $\tau_{t}^{2}=0.001$ for each t . To assess model performance with different degrees of spatial correlation in the risk surface, we generate spatially correlated random effects $\phi_{t}$ where the spatial dependence parameters are varied over $\rho_{s},\rho_{s_{t}}=0.9,0.6,0.3,0$ . Here a value of 0.9 corresponds to strong spatial dependence, values of (0.6, 0.3) correspond to moderate and weak dependence, respectively, while a value of 0 corresponds to spatial independence.

Clustering is induced into these spatial surfaces by the mean of the multivariate Gaussian distribution used to generate $\phi_{t}$ , which we denote by $\mu_{t}=(\mu_{1t},\ldots ,\mu_{nt})$ . At each time period we consider high, medium and low risk levels, which are generated by specifying a piecewise constant mean function so that each $\mu_{it}\in \{-Z,0,Z\}$ . Thus geographically neighbouring areal units that have the same mean value are in the same cluster as their disease risks will be similar, while those pairs that have different values will exhibit a step-change in their risks and hence are in different clusters. The value of Z is varied in the different scenarios of our simulation design as either Z=0.5 or Z=1 , which, respectively, correspond to small and large differences in disease risk between neighbouring areal units in different clusters. We consider two cases for this clustering, which, respectively, favour variants A (Case 1) and B (Case 2) of our model.

- **Case 1** – the simulated clusters remain constant during the study period, which is achieved by setting $\mu_{it}=\mu_{il}$ for all t≠l .
- **Case 2** – the simulated clusters evolve over time, which is achieved by $\mu_{it}\neq \mu_{il}$ for some pairs of time periods (t,l) .

**Figure 2.**
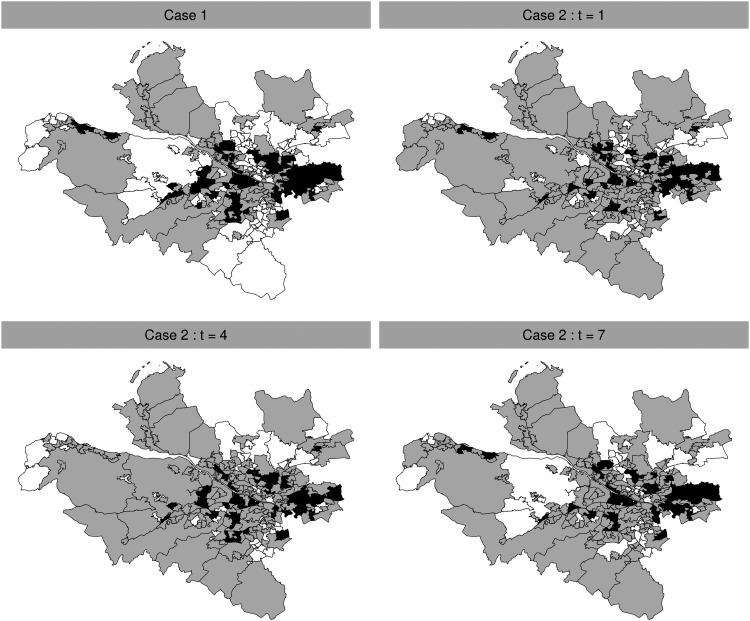
Maps of the simulated cluster structures under Case 1 (top-left) and Case 2 (top-right and bottom). High-risk, medium-risk and low-risk clusters are, respectively, shaded in black, grey and white.

Figure 2 provides the maps of the cluster structures simulated for both Case 1 and Case 2, where areal units in the high-risk, medium-risk and low-risk clusters are, respectively, shaded in black, grey and white. Under Case 1, the simulated clusters are common to all time periods, while under Case 2, the cluster structures at time periods t=1,4,7 are shown here. The remaining structures for other time periods are presented in Section 2 of the Supplemental Material. For Case 2, the temporal evolution of the clusters is achieved by randomly selecting a small number of areal units and changing their cluster membership for each time period. The chosen cluster structure template is based on the motivating study, by partitioning the set of IZs into three groups based on their SIR values. Finally, we also consider a scenario where Z=0 , which corresponds to a spatially smooth risk surface with no clusters for each time period. Note, in this case as there are no clusters in the simulated risk surfaces then there is no difference between Case 1 and Case 2. Our simulation study thus has 30 sub-scenarios in its design, which are summarised in Table 2. Thus in this study we vary the following quantities: (i) constant and time-varying clusters via Case 1 and Case 2; (ii) varying cluster magnitudes via Z=1,0.5,0 ; (iii) varying disease prevalences via SF=1,2,4 ; and (iv) varying levels of spatial autocorrelation via $\rho_{s},\rho_{s_{t}}=0.9,0.6,0.3,0$ .

**Table 2.** Description of the scenarios in the simulation study. SF indicates the scale factor used for the expected values.

Cluster cases	Z	SF	$\left(\rho_{s},\rho_{s_{t}}\right)$
Case 1/2	Z∈{1,0.5}	SF∈{1,2,4}	$\rho_{s},\rho_{s_{t}}=0.9$
–	Z=0	SF∈{1,2,4}	$\rho_{s},\rho_{s_{t}}=0.9$
Case 1/2	Z∈{1,0.5}	SF=1	$\rho_{s},\rho_{s_{t}}\in \{0.6,0.3,0\}$
–	Z=0	SF=1	$\rho_{s},\rho_{s_{t}}\in \{0.6,0.3,0\}$

### 4.2 Results

One hundred simulated data sets are generated under each of the 30 scenarios shown in Table 2, and the five models **ST-A**, ST-A*, **ST-B**, ST-B* and **ST-N** are fitted to each data set. In all cases, inference is based on a single Markov chain with 100,000 MCMC samples, 80,000 of which were discarded for the burn-in period and the remaining 20,000 samples were thinned by 10 to reduce their autocorrelation. The main results are shown below, while the sensitivity of these results to the choice of prior distribution is assessed in Section 3 of the Supplemental Material. Specifically, we consider two commonly used alternatives to the Inverse-Gamma (1,0.01) prior distribution for $\tau_{t}^{2}$ used here (see Anderson et al.^13^ and Spiegelhalter et al.^29^), which are Inverse-Gamma (0.001,0.001) and Inverse-Gamma (0.5,0.0005) , and the results show almost no sensitivity to these choices of prior distribution.

The results of the main study are shown in Tables 3 and 4, which summarise the modelling performance using three metrics. The accuracy of disease risk estimation is quantified by the root mean square error (RMSE) of the risk estimates and the corresponding coverage probabilities of the 95% credible intervals. The correctness of the estimated cluster structures is measured by the adjusted Rand index^30^ between the true and estimated cluster structures over all time periods. For this metric a value of 1 indicates complete agreement between two cluster structures, a value of 0 indicates that the data points are randomly allocated to the two cluster structures, and a value less than 0 indicates that the level of agreement between the two cluster structures is smaller than that arising from randomly allocated data points. Table 3 displays these performance metrics for each model under different scenarios of Cases 1/2, Z=1,0.5,0 and SF=1,2,4 , but in this table, the spatial random effects are simulated under the strong spatial dependence scenario with $\rho_{s},\rho_{s_{t}}=0.9$ .

**Table 3.** Median values of the RMSE, 95% credible interval coverages and adjusted Rand Index (ARI) for each model and scenario. In the scenarios considered here $\rho_{s}=\rho_{s_{t}}=0.9$ is used to simulate the spatial random effects for each time period.

Performance metric	Cluster case	*Z*	SF	Model				
				**ST-A**	ST-A*	**ST-B**	ST-B*	**ST-N**
RMSE	**Case 1**	1	1	0.087	0.068	0.091	0.074	1.175
		0.5	1	0.071	0.060	0.099	0.096	0.119
		1	2	0.115	0.094	0.130	0.116	1.155
		0.5	2	0.097	0.082	0.159	0.158	0.157
		1	4	0.150	0.128	0.207	0.192	1.109
		0.5	4	0.151	0.120	0.225	0.224	0.204
	**Case 2**	1	1	0.135	0.132	0.091	0.074	0.904
		0.5	1	0.114	0.111	0.101	0.099	0.113
		1	2	0.188	0.181	0.142	0.126	0.864
		0.5	2	0.153	0.148	0.155	0.155	0.150
		1	4	0.267	0.249	0.217	0.209	0.817
		0.5	4	0.204	0.196	0.218	0.217	0.195
	**–**	0	1	0.025	0.034	0.083	0.070	0.026
		0	2	0.029	0.040	0.112	0.095	0.028
		0	4	0.091	0.049	0.145	0.131	0.033
Coverage probability	**Case 1**	1	1	0.975	0.975	0.969	0.955	0.801
		0.5	1	0.974	0.974	0.928	0.920	0.951
		1	2	0.979	0.973	0.948	0.931	0.805
		0.5	2	0.972	0.974	0.843	0.842	0.949
		1	4	0.981	0.969	0.907	0.897	0.843
		0.5	4	0.937	0.959	0.777	0.764	0.947
	**Case 2**	1	1	0.935	0.942	0.964	0.969	0.811
		0.5	1	0.937	0.930	0.900	0.884	0.950
		1	2	0.915	0.938	0.921	0.923	0.851
		0.5	2	0.928	0.913	0.827	0.819	0.948
		1	4	0.862	0.929	0.885	0.860	0.881
		0.5	4	0.901	0.878	0.783	0.785	0.942
	**–**	0	1	0.991	0.993	0.694	0.905	0.996
		0	2	0.992	0.997	0.759	0.893	0.998
		0	4	0.979	0.998	0.829	0.891	0.999
Adjusted Rand Index (ARI)	**Case 1**	1	1	1	1	0.986	0.976	–
		0.5	1	1	1	0.843	0.853	–
		1	2	1	1	0.891	0.893	–
		0.5	2	0.983	1	0.514	0.589	–
		1	4	1	1	0.740	0.787	–
		0.5	4	0.617	0.937	0.319	0.367	–
	**Case 2**	1	1	0.353	0.409	0.987	0.987	–
		0.5	1	0	0.412	0.711	0.655	–
		1	2	0.351	0.393	0.608	0.907	–
		0.5	2	0	0.360	0.383	0.426	–
		1	4	0.359	0.395	0.504	0.600	–
		0.5	4	0	0.290	0.235	0.267	–
	**–**	0	1	1	0	0	0	–
		0	2	1	0	0	0	–
		0	4	0	0	0	0	–

**Table 4.** Median values of the RMSE, 95% credible interval coverages and adjusted Rand Index (ARI) for each model and scenario. In the scenarios considered here the expected counts from the motivating application are used to generate disease data.

Performance metric	Cluster case	*Z*	$\rho_{s}/\rho_{s_{t}}$	Model				
				**ST-A**	ST-A*	**ST-B**	ST-B*	**ST-N**
RMSE	**Case 1**	1	0.9	0.087	0.068	0.091	0.074	1.175
		0.5	0.9	0.071	0.060	0.099	0.096	0.119
		1	0.6	0.088	0.068	0.090	0.074	1.172
		0.5	0.6	0.076	0.059	0.097	0.094	0.117
		1	0.3	0.089	0.071	0.092	0.077	1.191
		0.5	0.3	0.100	0.061	0.099	0.095	0.118
		1	0	0.094	0.078	0.097	0.084	1.246
		0.5	0	0.102	0.064	0.101	0.100	0.117
	**Case 2**	1	0.9	0.135	0.132	0.091	0.074	0.904
		0.5	0.9	0.114	0.111	0.101	0.099	0.113
		1	0.6	0.134	0.128	0.089	0.075	0.892
		0.5	0.6	0.114	0.110	0.102	0.100	0.113
		1	0.3	0.131	0.128	0.092	0.076	0.868
		0.5	0.3	0.116	0.113	0.101	0.101	0.115
		1	0	0.134	0.129	0.094	0.081	0.884
		0.5	0	0.116	0.112	0.105	0.103	0.115
	**–**	0	0.9	0.025	0.034	0.083	0.070	0.026
		0	0.6	0.026	0.030	0.084	0.071	0.024
		0	0.3	0.029	0.032	0.085	0.071	0.026
		0	0	0.035	0.038	0.085	0.073	0.033
Coverage probability	**Case 1**	1	0.9	0.979	0.975	0.969	0.955	0.801
		0.5	0.9	0.974	0.974	0.928	0.920	0.951
		1	0.6	0.978	0.971	0.969	0.954	0.791
		0.5	0.6	0.973	0.972	0.930	0.925	0.952
		1	0.3	0.978	0.968	0.968	0.952	0.797
		0.5	0.3	0.947	0.969	0.922	0.921	0.949
		1	0	0.974	0.946	0.964	0.934	0.781
		0.5	0	0.947	0.950	0.909	0.899	0.949
	**Case 2**	1	0.9	0.935	0.942	0.964	0.969	0.811
		0.5	0.9	0.937	0.930	0.900	0.884	0.950
		1	0.6	0.932	0.943	0.961	0.967	0.809
		0.5	0.6	0.935	0.929	0.898	0.889	0.949
		1	0.3	0.934	0.943	0.957	0.962	0.810
		0.5	0.3	0.936	0.930	0.900	0.894	0.949
		1	0	0.936	0.943	0.951	0.941	0.793
		0.5	0	0.935	0.930	0.877	0.869	0.949
	**–**	0	0.9	0.991	0.993	0.694	0.905	0.996
		0	0.6	0.987	0.997	0.710	0.884	0.998
		0	0.3	0.977	0.995	0.683	0.885	0.996
		0	0	0.934	0.984	0.705	0.861	0.982
Adjusted Rand Index (ARI)	**Case 1**	1	0.9	1	1	0.986	0.976	–
		0.5	0.9	1	1	0.843	0.853	–
		1	0.6	1	1	0.985	0.975	–
		0.5	0.6	0.992	1	0.847	0.846	–
		1	0.3	1	1	0.985	0.975	–
		0.5	0.3	0.541	1	0.846	0.856	–
		1	0	1	1	0.988	0.976	–
		0.5	0	0.541	1	0.828	0.839	–
	**Case 2**	1	0.9	0.353	0.409	0.987	0.987	–
		0.5	0.9	0	0.412	0.711	0.655	–
		1	0.6	0.367	0.412	0.987	0.988	–
		0.5	0.6	0	0.411	0.645	0.699	–
		1	0.3	0.358	0.409	0.980	0.987	–
		0.5	0.3	0	0.388	0.691	0.735	–
		1	0	0.357	0.397	0.981	0.987	–
		0.5	0	0	0.387	0.618	0.687	–
	**–**	0	0.9	1	0	0	0	–
		0	0.6	1	0	0	0	–
		0	0.3	1	0	0	0	–
		0	0	1	0	0	0	–

The non-cluster model **ST-N** mainly performs poorly in terms of risk estimation compared to the clustering models proposed here, having mostly larger RMSE values and coverage probabilities as low as 0.8. An exception to this is when there are no clusters in disease risk ( Z=0 ), and in this case the **ST-N** model performs similarly to the constant cluster models **ST-A** and ST-A*. The other scenario in which **ST-N** performs comparably to the cluster models is when the disease is rare (SF = 4) and the cluster boundaries are small in magnitude ( Z=0.5 ), which is because in this scenario the clusters are hard to identify based on their small size and small number of disease cases.

The four clustering models perform best when the disease prevalence is high (SF = 1) and the clusters are large in size ( Z=1 ) as expected, which is because in these scenarios there are more disease cases from which to identify more prominent clusters. In this situation, the RMSE values are very low compared to the range of the true risks, coverage probabilities are close to their nominal 0.95 levels, and cluster identification is very good with ARI values either very close to or equal to one. When the disease prevalence decreases (SF > 1) and the clusters are less pronounced the models perform less well, but the ARI values are still relatively close to one in most scenarios unless the disease is rare (SF = 4) and the clusters are small in magnitude ( Z=0.5 ). Additionally, the models identify temporally static clusters better than temporally varying ones, as the results for models **ST-A** and ST-A* in Case 1 are generally better than those for models **ST-B** and ST-B* in Case 2. This improved performance in the static cluster case is because the temporal replication in the true cluster structure yields more data from which to identify clusters, thus resulting in improved model performance.

When the clusters are temporally constant (Case 1) then as expected models **ST-A** and ST-A*, which assume static clusters, produce more accurate risk estimates (lower RMSE) than model **ST-B** and ST-B* in all cases, as well as producing adjusted Rand indices that are generally very close to one. Similarly, when the clusters evolve over time (Case 2) then as expected models **ST-B** and ST-B*, which allow for dynamic clusters, perform better than **ST-A** and ST-A* in most scenarios. The only slight exception to this is when the disease is rare (SF = 4) and the clusters are not large in size ( Z=0.5 ), which is the case where the clusters are hardest to identify and hence all the cluster models perform less well (small ARI values). Estimating $\left(\rho_{s},\rho_{s_{t}}\right)$ (ST-A*, ST-B*) rather than fixing them at 0.99 (**ST-A**, **ST-B**) produces better results overall, with lower RMSE values and higher ARI values in almost all scenarios.

Table 4 summarises model performance under different levels of spatial autocorrelation (values of $\rho_{s},\rho_{s_{t}}$ ), and the results are based on the expected disease counts from the motivating data ( SF=1 ). The results show that reducing the spatial dependence in the data does not seem to have any substantial effect on the ability of the proposed cluster models to estimate disease risk or identify the correct cluster structure, as the ARI results do not differ greatly as the spatial dependence parameters $\left(\rho_{s},\rho_{s_{t}}\right)$ vary. Additionally, the coverage probabilities are also largely unaffected by this change, and the RMSE values increase very slightly (suggesting worse estimation) as $\left(\rho_{s},\rho_{s_{t}}\right)$ gets closer to zero, due to a reduction in the borrowing of strength spatially when doing the estimation. In Case 1, ST-A* again outperforms **ST-A** across the board, suggesting that estimating $\rho_{s}$ leads to better model performance regardless of the level of spatial autocorrelation in the data. This effect is also seen when comparing ST-B* and **ST-B** in Case 2, in terms of RMSE, but for the ARI results the two models are very similar. Finally, the globally smooth non-clustering model **ST-N** again performs uniformly badly when there are clusters in the data ( Z>0 ) as expected.

Finally, we also tested the performance of the proposed models under a set of model-free scenarios. In these scenarios, we fix the risks of the areas with high, medium and low risk levels at constant values of {exp(Z),1,exp(−Z)} , respectively, rather than generating the risks by simulating spatial random effects using a multivariate Gaussian distribution with a Leroux covariance structure as described in Section 4.1. In this model free case, the proposed models still provide robust and reasonable results in terms of estimating risk and cluster structure, and to conserve space the results are presented in Section 4 of the Supplemental Material.

## 5 Results from the Greater Glasgow respiratory disease study

### 5.1 Model fitting and inference

The four spatio-temporal models **ST-A**, ST-A*, **ST-B** and ST-B* proposed in Section 3 are applied to the Greater Glasgow respiratory disease study introduced in Section 2.1, with the aims of estimating the spatio-temporal patterns in disease risk and identifying the locations of any high and low risk clusters. Model **ST-N** without clustering is also fitted to the data, to observe how its model fit compares with the clustering models proposed here. In all cases covariate information is not included in the models, because our aim is to identify clusters in the risk surface rather than clusters in the residual risk surface after covariate adjustment. Posterior inference for all models is based on 10 independent Markov chains, where each chain is run for 100,000 samples. The first 80,000 samples from each chain are removed as the burn-in period and the remaining samples are thinned by 10, which yields a total of 20,000 samples across all 10 chains. These MCMC samples were deemed to have converged, which was assessed both by examining parameter trace plots and by Geweke^31^ diagnostics. The computational time required to fit each model is outlined in Section 5 of the Supplemental Material.

### 5.2 Overall model fit

The overall fit of each model is summarised in Table 5 by the deviance information criterion (DIC^32^) and the effective number of independent parameters ( $p_{d}$ ). The table shows that the four clustering models proposed here fit the data better than the non-clustering model **ST-N**, as the latter has a DIC value that is higher by between 0.7% and 4.9% than those from the clustering models. The time-varying clustering models **ST-B** and ST-B* have lower DIC values than the static clustering models **ST-A** and ST-A*, while allowing $\left(\rho_{s},\rho_{s_{t}}\right)$ to be estimated (ST-A*, ST-B*) rather than fixed at 0.99 (**ST-A**, **ST-B**) also produces a better model fit. Additionally, our models are also preferred because they model the data using fewer effective parameters, with reductions in $p_{d}$ varying between 16.0% and 33.6% compared to **ST-N**. This reduced effective number of parameters is due to a reduction in the spatial random effects variance $\tau_{t}^{2}$ for the cluster models, which can be seen clearly in Table 6. This reduction in the random effects variation is because by our approach the spatial random effects are only forced to smooth towards their neighbours in the same cluster, that is, those neighbours that have similar random effects values. In contrast, in model **ST-N** this smoothing is with all neighbouring areas, even those that have very different random effect values which hence inflates the variance.

**Table 5.** Deviance information criterion (DIC) and the effective number of independent parameters ( $p_{d}$ ) for each model.

	**ST-A**	ST-A*	**ST-B**	ST-B*	**ST-N**
DIC	14 631	14 587	14 272	14 040	14 730
$p_{d}$	1 344	1 362	1 268	1 183	1 580

**Table 6.** Summary of the estimated number of clusters with 95% credible intervals and spatial random effects variance $\tau_{t}^{2}$ at each time period.

	Time period						
	t=1	t=2	t=3	t=4	t=5	t=6	t=7
clusters						
**ST-A**	2 (1, 3)	2 (1, 3)	2 (1, 3)	2 (1, 3)	2 (1, 3)	2 (1, 3)	2 (1, 3)
ST-A*	2 (2, 5)	2 (2, 5)	2 (2, 5)	2 (2, 5)	2 (2, 5)	2 (2, 5)	2 (2, 5)
**ST-B**	4 (2, 7)	5 (3, 9)	4 (4, 9)	4 (3, 8)	4 (2, 7)	3 (3, 8)	4 (2, 7)
ST-B*	5 (4, 6)	6 (3, 8)	6 (4, 8)	5 (4, 8)	5 (3, 5)	5 (3, 7)	5 (4, 9)
**ST-N**	–	–	–	–	–	–	–
$\tau_{t}^{2}$							
**ST-A**	0.056	0.088	0.069	0.083	0.084	0.083	0.075
ST-A*	0.061	0.081	0.069	0.077	0.078	0.081	0.075
**ST-B**	0.006	0.004	0.004	0.004	0.003	0.004	0.004
ST-B*	0.004	0.003	0.003	0.003	0.003	0.003	0.003
**ST-N**	0.265	0.291	0.292	0.268	0.254	0.283	0.297

### 5.3 Temporal trends in disease risk

**Figure 3.**
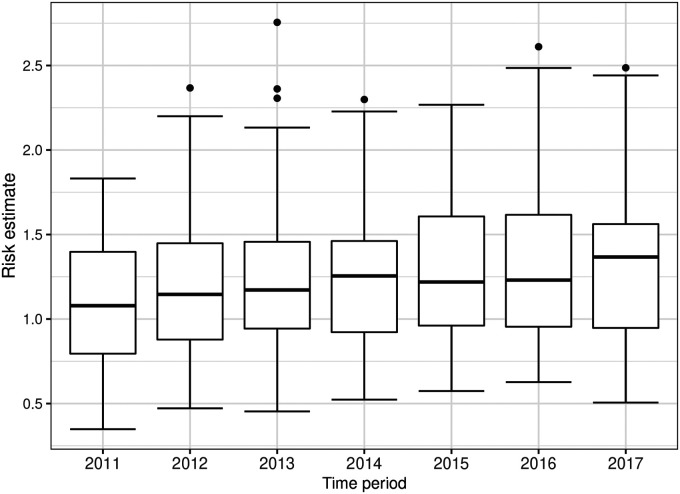
Boxplots of the risk estimates (posterior median) from model ST-B* for all the areal units over time.

We present the estimated temporal trends in disease risk from model ST-B* in Figure 3, because it is the best fitting model in terms of DIC. However, we note that the results from the other models are similar, as the mean absolute differences in the posterior median risk estimates between each pair of models range between 0.009 and 0.083 over all years and IZs. Figure 3 displays the boxplots of the risk estimates from all the areal units over time, and shows a generally increasing trend in risk. These risk estimates are relative to the expected number of hospitalisations computed using national respiratory hospitalisation rates for the whole of Scotland between 2011 and 2017, because national rather than Greater Glasgow rates are used by Public Health Scotland when quantifying disease risk. In 2011, the average risk across Greater Glasgow is 1.10, suggesting that on average respiratory disease risk in Greater Glasgow is about 10% higher than the Scottish average. This rises to 1.28 in the final year 2017, which is thus 28% higher than the overall Scotland average. Thus an elevated risk is observed in Glasgow for the entire time period of this study, which corroborates the well-known *Glasgow effect*^33^ which is the phenomenon that Glasgow exhibits some of the poorest health in western Europe.

### 5.4 Spatio-temporal cluster structure

The previous section highlighted that the risk estimates from the clustering and non-clustering models are similar, and so the main advantage of using the clustering models is the additional inference they provide on the locations of clusters of areas that exhibit elevated or reduced disease risks compared to their neighbours. The top panel of Table 6 displays the posterior mode for the number of clusters estimated by each cluster model for each year, with uncertainty measured by the 95% credible intervals. If there are multiple modes present in the Markov chain, then the one yielding the fewer effective number of parameters is chosen in the interests of model parsimony. Note that by clusters we mean the number of non-spatial clusters (distinct risk levels) that correspond to the posterior mode of $\left(\tilde{W},{\tilde{W}}_{t}\right)$ , rather than the number of spatially distinct clusters observable on the risk maps presented below. Models **ST-A** and ST-A* have selected the same cluster structure with two distinct clusters or risk levels, and that by design this structure is common to all time periods. In contrast, model **ST-B** identifies between three and five different cluster levels depending on the year, although most years have four different clusters, and model ST-B* detects five distinct clusters for most years. We now present the estimated cluster structures from the models, focusing on both the posterior mode clusters that are static (ST-A*) and dynamic (ST-B*) over time, as well as illustrating posterior uncertainty in the cluster structures for ST-A*.

#### 5.4.1 Static and time-varying clusters based on the posterior mode cluster structure

**Figure 4.**
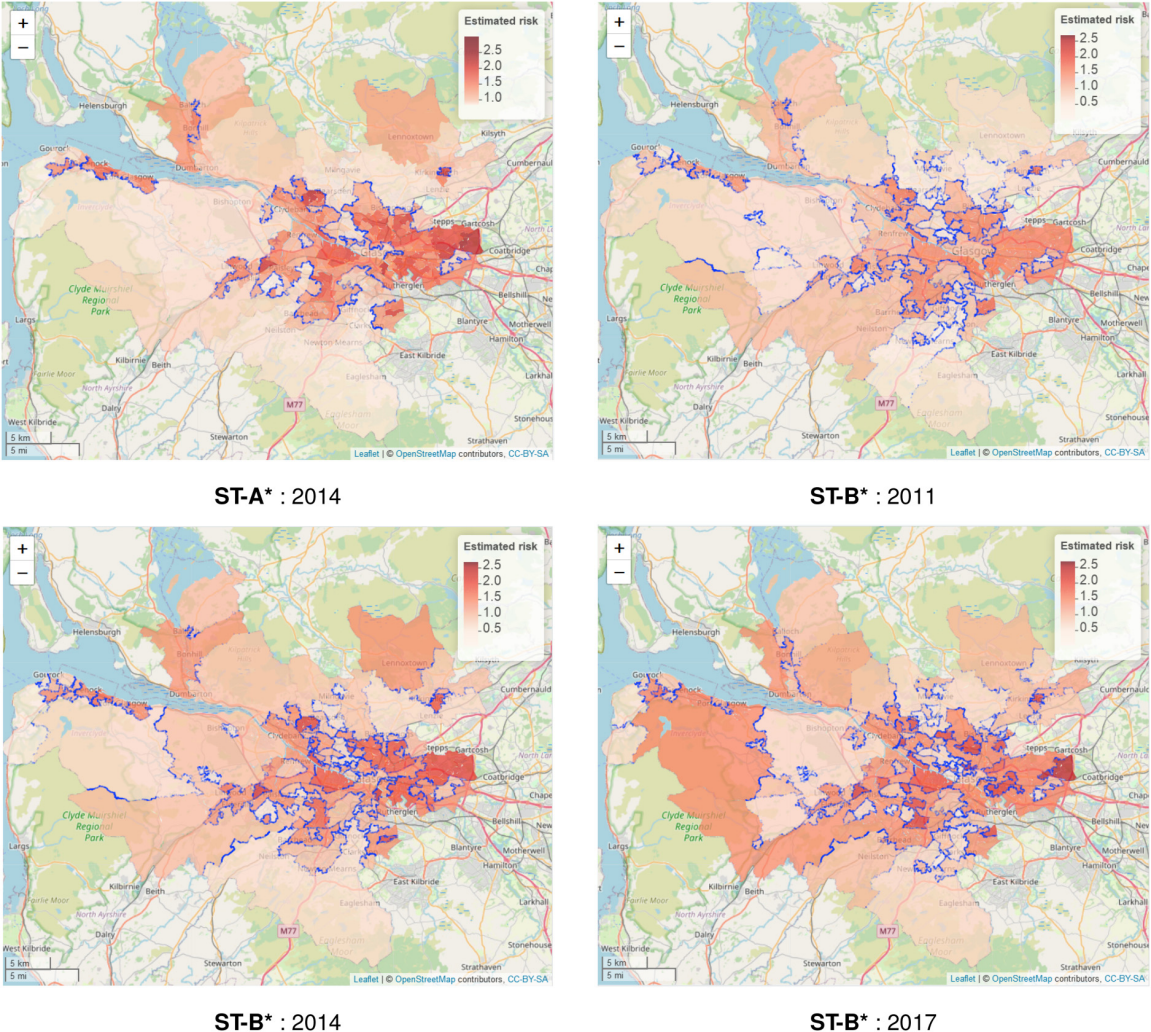
Maps of the disease risk estimates (posterior median) in Greater Glasgow for 2011, 2014 and 2017 from models ST-A* and ST-B*. The dots on the map indicate the identified discontinuities, which are determined using the posterior mode of $\left(\tilde{W},{\tilde{W}}_{t}\right)$ .

As Table 5 shows that models ST-A* and ST-B* have lower DIC values than models **ST-A** and **ST-B**, we present these results here. The top left panel in Figure 4 displays the estimated spatial pattern in disease risk from model ST-A* for 2014, which is chosen because it is the middle year of our study. The blue dots relate to the cluster boundaries as defined by the posterior mode of $\tilde{W}$ , which in this static clustering case are common to all time periods. The remaining three panels of the figure present the estimated risk surfaces from model ST-B* for 2011, 2014 and 2017, the first, middle and last years of the study period, respectively. In these cases, the clusters/discontinuities denoted by blue dots vary over time and are specific to the year in question, and are again determined by the posterior mode of ${\tilde{W}}_{t}$ . These cluster boundaries (discontinuities) represent two IZs that are geographically adjacent but are in different clusters, suggesting they have substantially different risks. For completeness, estimated risk patterns and clusters for all time periods from models ST-A* and ST-B* are presented in Section 6 of the Supplemental Material.

The figure shows there are a number of similarities between the selected cluster structures from the two cluster models, with the same areas being identified as having very different risks compared to their neighbours. For example, both models identify the large high-risk cluster in the East End of Glasgow (far east of the map), which contains a number of socio-economically deprived areas such as Easterhouse and Barlanark. Additionally, the models identify a cluster of areas to the north of the city containing Springburn and Summerston, as well as another along the southern bank of the River Clyde including Govan and Hutchesontown. They also pick out some high risk areas in the north west including the deprived areas of Drumchapel and Drumry, which are bordered to the north by the more affluent and low-risk Bearsden area. Additionally, another large region of low risk areas identified by both models is the affluent West End of the city such as Dowanhill, which is just to the south of Bearsden. Note, as all the clustering methods were used to adjust the border sharing neighbourhood matrix, clusters cannot be found between areas on opposite banks of the river Clyde, which runs north-west through the study region.

In addition, there is some evidence of a changing cluster structure over time estimated by model ST-B* that is worth noting. For example, the large rural areas of Inverclyde in the far west of the study region exhibit low risks in 2011, whereas by 2017 they have joined a moderately high risk cluster. However, comparing the clustering results from the two modelling approaches we find that while ST-B* is very flexible in capturing the temporal evolution of clusters, it can also be susceptible to identifying discontinuities (clusters) caused by random noise that are present for some years but not for others, which thus make the interpretation of an evolving cluster structure less clear cut. This happens because in the ST-B* model each candidate cluster structure in stage one is elicited using data for a single year, which thus could be affected by random noise in the data. However, model ST-A* is less vulnerable to this phenomenon, because the clustering is applied to the data from multiple time periods. Thus despite model ST-A* having a higher DIC than model ST-B*, its consistency of clustering may lead to robust and reliable clusters, as can be visually observed in Figure 4.

#### 5.4.2 Posterior uncertainty in the estimated cluster structure

**Figure 5.**
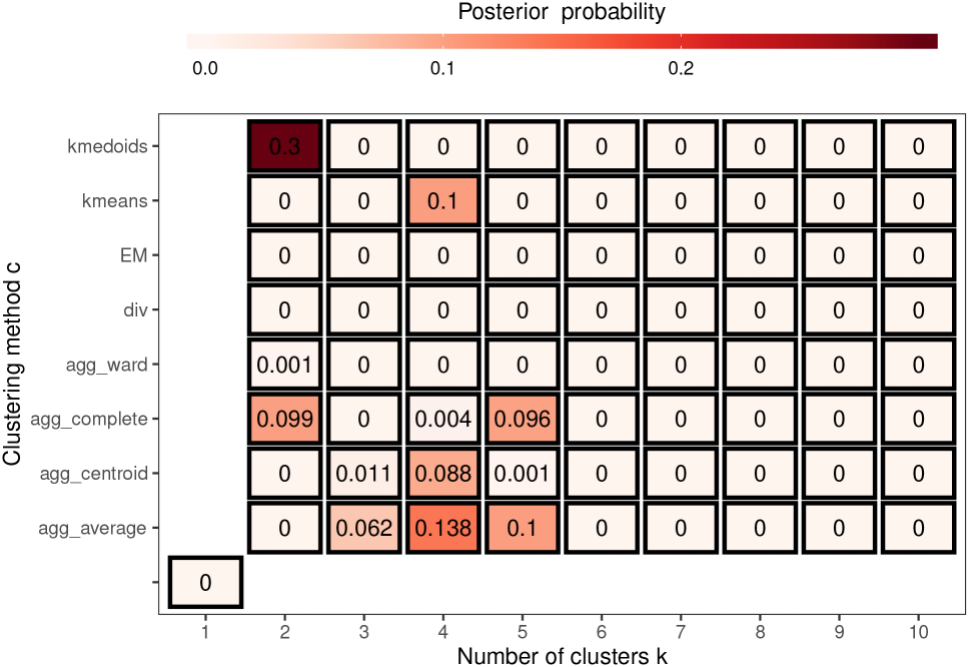
Summary of the posterior distribution of $\tilde{W}$ over 10 Markov chains from model ST-A*.

**Figure 6.**
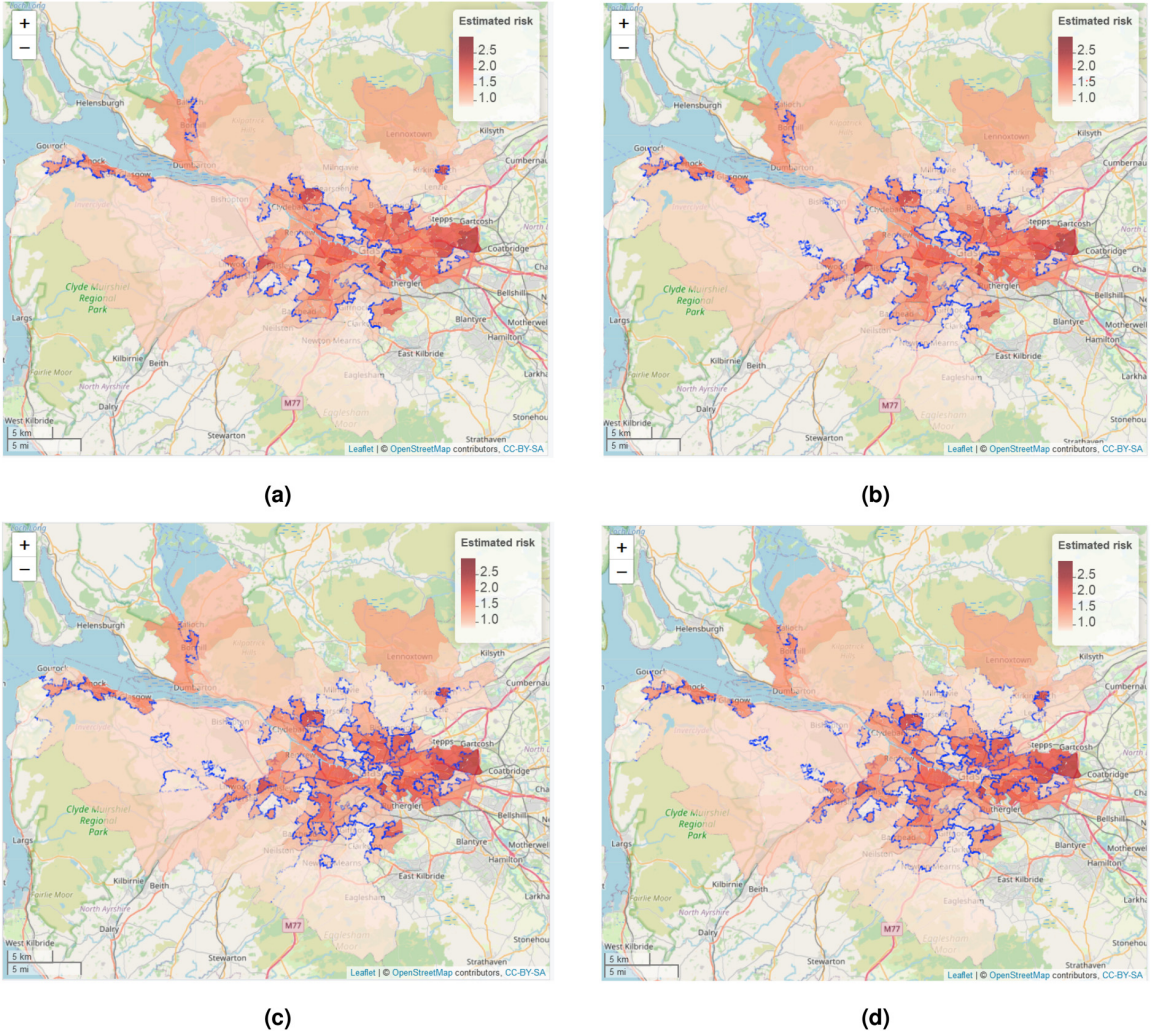
The four most likely cluster structures selected by model ST-A*, which are represented by blue dots. The colour shading for the areas denotes posterior median disease risk in 2014 (the middle of the study period). The colour version of the figure is available online.

To illustrate the uncertainty in the estimated cluster structure Figure 5 summarises the posterior distribution of $\tilde{W}$ obtained from the 10 Markov chains for model ST-A*, which assumes a single cluster structure for all time periods. In the figure each grid square represents a candidate neighbourhood matrix $W^{\left(c,k\right)}$ corresponding to a distinct cluster structure, where the horizontal axis denotes the number of clusters and the vertical axis denotes the clustering method. Note that the grid square on the bottom left corner corresponds to the border sharing $W=W^{\left(1,1\right)},\ldots ,W^{\left(M,1\right)}$ (i.e. k=1 ) which represents no clusters in disease risk. The figure shows that this no cluster solution is not supported by the data, with a posterior probability of zero. The figure also shows that the posterior distribution is mainly centred on eight different cluster structures, which each have posterior probabilities above 0.06. The top four of these cluster structures have posterior probabilities of 0.3, 0.138, 0.1 and 0.1 respectively, and are displayed in Figure 6 and denoted by (a), (b), (c) and (d). Cluster structure (a), which has the highest posterior probability, identifies 2 spatial clusters (risk levels), although from the map it is clear that this corresponds to many more spatially distinct clusters. In contrast, structures (b) to (d) identify 4 or 5 distinct cluster levels, which is why there are more spatially distinct clusters identified in panels (b) to (d) in the figure. The adjusted Rand Index values between these four cluster structures range between 0.49 and 0.77, suggesting moderate agreement between them. The figure shows that all four cluster structures appear to mostly correspond to sizeable changes in disease risk between adjacent IZs, suggesting that the clustering model can identify such spatially distinct clusters. To conserve space, the posterior distribution of ${\tilde{W}}_{t}$ for model ST-B* is presented in Section 7 of the Supplemental Material.

## 6 Discussion

Smoothing models based on geographical adjacency are commonly used to estimate risk in disease mapping studies, and they force geographical neighbours to have similar disease risks. However, this smoothing will mask any discontinuities present in the risk surface, leading to sub-optimal risk estimation and no cluster identification. The latter is important because health agencies often target additional resources at communities with the greatest need, thus they need to identify the spatial extent of a cluster of high-risk areas. This paper has proposed a novel clustering-based adjacency modelling approach in the spatio-temporal domain, which can simultaneously estimate disease risk and identify the locations of clusters of high/low risk areas that may be static or evolve dynamically over time. Our methodology first constructs a large collection of candidate cluster structures for the data, which each corresponds to a candidate neighbourhood matrix. Then a spatio-temporal model is fitted to the data that jointly estimates disease risk and the cluster structure, the latter by treating the neighbourhood matrix as a parameter to be estimated from the set of candidate structures constructed in stage 1. As this matrix determines the spatial correlation structure in the data, our approach extends the standard practice in areal unit modelling that naively assumes the geographical adjacency neighbourhood matrix provides a suitable spatial correlation structure for the data. In fact, our methodology parallels the standard practice in geostatistics, where an appropriate spatial dependence structure is identified for the data (e.g. by variogram analysis) rather than a single structure being assumed without assessing its suitability.

Existing cluster based methods^15,14^ fit a separate model to each candidate cluster structure, and identify the optimal cluster structure by model comparison approaches. In contrast, our approach has the advantages of having substantially reduced computational time (it only fits a single spatio-temporal model), and allowing the uncertainty in the cluster structure to be quantified when estimating disease risk in the second step. For example, although the computational time of model ST-A* is almost 60% longer than that of the same model with a fixed neighbourhood matrix (or cluster structure), the latter would have to be fitted 73 times in a model comparison setting because we have 73 candidate cluster structures to consider. One approach that does allow for cluster uncertainty is Anderson et al.,^15^ and we have extended this approach by considering a spatio-temporal rather than a spatial domain and proposing cluster models where the spatial clusters either remain fixed or evolve dynamically over time. The simulation study shows that our models provide accurate risk estimates in the presence of clusters (discontinuities), particularly performing better than a similar non-cluster model. This improved performance is because our models account for the clusters in risk by estimating an appropriate neighbourhood matrix, which better represents the spatial autocorrelation structure in the data, therefore removing any redundant smoothing of the spatial random effects between neighbours. The study also shows that our models can accurately identify both static and temporally dynamic clusters, with high ARI values being obtained in both cases. However, as expected cluster identification is more accurate for static rather than dynamic clusters, which is due to the former having more data with which to identify the high-risk clusters due to them recurring for multiple time periods.

We have used a range of classical clustering techniques to identify the candidate cluster structures rather than scan statistics,^10,34,35^ because the latter only identify a relatively small number of clusters of areas exhibiting high-risks rather than partitioning the risk surface into different risk levels, which is required here for constructing candidate neighbourhood matrices. In our method, the maximum number of clusters K denotes the maximum number of risk levels and not the maximum number of spatially distinct clusters, which is illustrated in Figure 4. In this paper, we have chosen K=10 , which has been shown to be a conservative (overly large) choice because the posterior distribution in Figure 5 has no posterior mass above five clusters. We note that the choice of K does not depend on the number of areas, because it does not relate in any way to the maximum number of spatially connected clusters that can be identified. Thus the factors to consider when choosing K is that if K is too small then the true cluster structure may not exist in the candidates, whereas if K is too large then the computational cost increases because longer MCMC runs are likely to be needed for convergence with such a large number of candidates.

The motivating application also illustrates the superiority of our clustering-based models compared to non-clustering alternatives, with our models able to produce a better model fit to the data and provide additional insight as to the locations of high-risk clusters. In regard to the latter, the majority of the identified clusters occur between geographical neighbours that exhibit very different disease risks, which will allow health agencies to better identify these high-risk areas and target additional resources where they are most needed. Each of our cluster models has its own appealing features, and the choice between them will depend on the aim of the analysis. The model with constant clusters over time may be more appropriate if the disease data have a high correlation in time and the main aim is to identify overall discontinuities for the entire study period. In contrast, if the disease data are less correlated in time and the cluster structures in particular years are of interest, then the model with temporally evolving clusters is likely to be the better choice. However, the latter model can sometimes pick out apparently erroneous clusters, due to the presence of random year to year fluctuations in the observed disease counts. Therefore in future work we will investigate a hybrid approach of the two considered here based on a 2q+1 years moving window, where the candidate cluster structures for a given year constructed in stage 1 are obtained by clustering the data for the year in question and the q years before and after. Additional potential extensions of our approach include adapting it for use with different spatio-temporal random effects structures, such as those of Knorr-Held^5^ and Rushworth et al.,^7^ as well as utilising it in the context of a spatio-temporal multivariate disease model, which allows for simultaneously estimating the risk of multiple diseases in space and time.

## Supplemental Material

sj-pdf-1-smm-10.1177_09622802221084131 - Supplemental material for Spatio-temporal disease risk estimation using clustering-based adjacency modelling Click here for additional data file. Supplemental material, sj-pdf-1-smm-10.1177_09622802221084131 for Spatio-temporal disease risk estimation using clustering-based adjacency modelling by Xueqing Yin, Gary Napier, Craig Anderson and Duncan Lee in Statistical Methods in Medical Research
