# Supplementary material for: Spatio-temporal disease risk estimation using clustering-based adjacency modelling
Source: Stat Methods Med Res. 2022 Mar 14;31(6):1184–203. doi: 10.1177/09622802221084131 (PMC9245163; doi:10.1177/09622802221084131)
Supplement: sj-pdf-1-smm-10.1177_09622802221084131 - Supplemental material for Spatio-temporal disease risk estimation using clustering-based adjacency modelling [file sj-pdf-1-smm-10.1177_09622802221084131.pdf]

Supplemental material to *Spatio-temporal disease risk estimation using clustering-based adjacency modelling*

1 Introduction

This supplemental material accompanies the paper entitled "Spatio-temporal disease risk estimation using clustering-based adjacency modelling" and has the following sections. Section 2 shows maps of the simulated cluster structures for each time period under Case 2 of the simulation study in the main paper. Section 3 performs a sensitivity analysis assessing the robustness of our methodology to changing the prior distribution for the spatial random effects variance parameter τ_t^2. In Section 4 we test the performance of the proposed models under a set of model-free scenarios, whereby the risk surface is assumed to be piecewise constant and not based on a set of spatially correlated random effects. Section 5 provides the computational time for the analysis of the motivating data under each model. Section 6 displays the estimated spatio-temporal risk patterns from 2011 to 2017 under the proposed cluster models **ST-A*** and **ST-B*** for the Glasgow respiratory disease data. Finally, the posterior distribution of $\widetilde{\boldsymbol{W}}_t$ for model **ST-B*** in the Glasgow respiratory disease study is shown in Section 7.

2 Simulated cluster structures for each time period under Case 2

Figure S1 presents maps of the simulated cluster structures for each time period under Case 2 (time-varying clusters) of the simulation study in the main paper, where high-risk, medium-risk and low-risk clusters are respectively shaded in black, gray and white. The figure shows that the cluster structure evolves slowly over time, which is realistic when studying a chronic rather than an infectious disease as is the case in the motivating study.

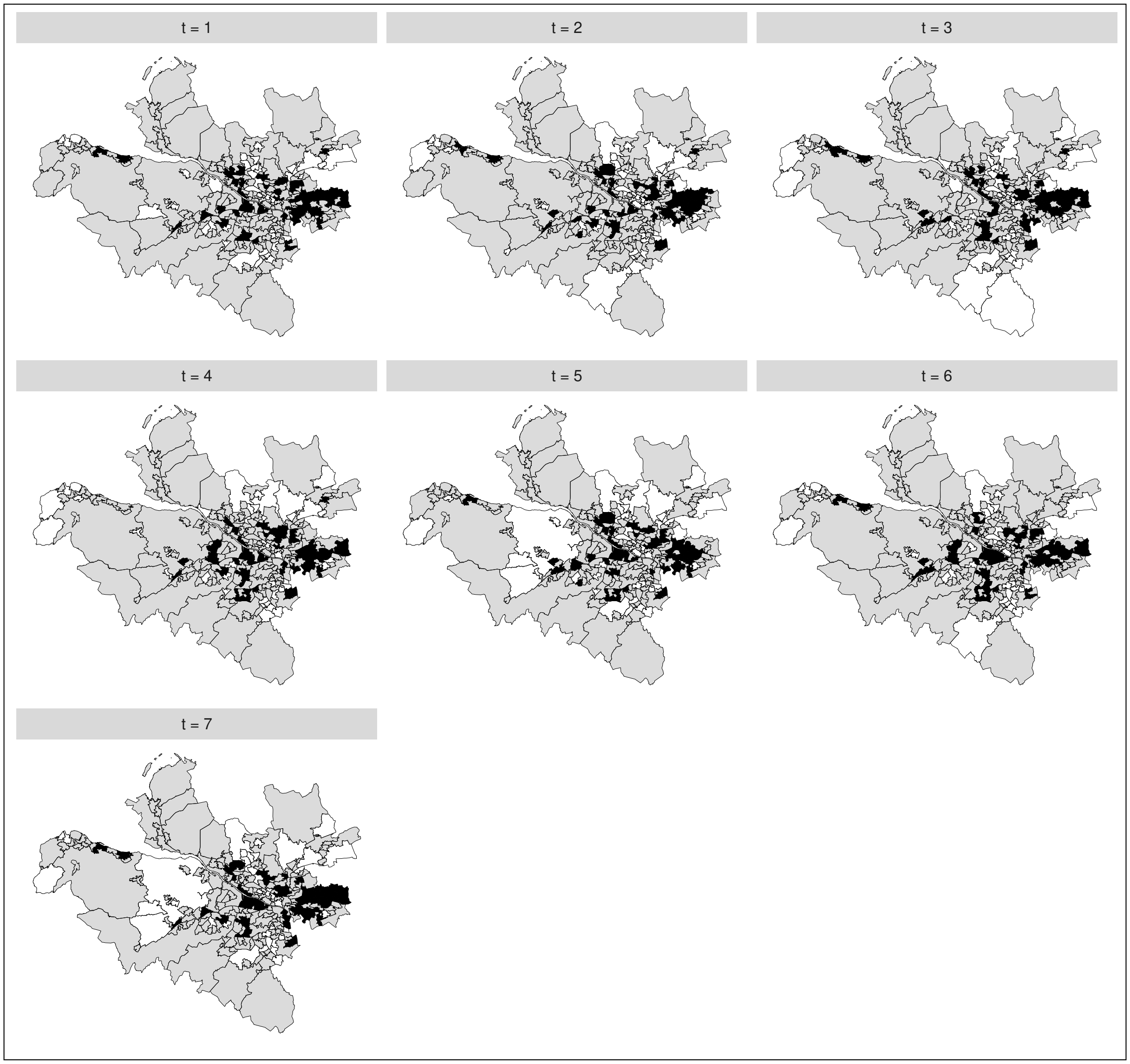

Figure S1: Maps of the simulated cluster structures for each time period under Case 2 of the simulation study in the main paper. High-risk, medium-risk and low-risk clusters are respectively shaded in black, gray and white.

3 Sensitivity analysis to changing the prior distribution for τ_t^2

In the main paper we use an Inverse-Gamma$(1, 0.01)$ prior for the spatial random effects variance τ_t^2 in the proposed models. To assess the impact of this prior for τ_t^2 on model performance, we re-run part of the

simulation study by fitting the proposed clustering models separately with both Inverse-Gamma$(0.001, 0.001)$ and Inverse-Gamma$(0.5, 0.0005)$ priors. Specifically, one hundred simulated data sets are generated as described in Section 4 of the main paper, where we consider $Z = 1, 0.5, 0$ and both Cases 1 (static clusters) and 2 (time-varying clusters). In generating the data we fix $\rho_s = \rho_{s_t} = 0.9$, and use the expected number of disease cases from the motivating data (i.e., SF = 1). The proposed models **ST-A**, **ST-B**, **ST-A*** and **ST-B*** are respectively applied to the data using the three different choices of prior Inverse-Gamma distribution for τ_t^2, and the results are summarised in Table S1.

The results show that changing the hyperparameters of the Inverse-Gamma prior for τ_t^2 does not seem to have any substantial effect on the ability of the proposed cluster models to estimate disease risk or identify the correct cluster structure, as the differences in RMSE, 95% coverage probabilities and ARI values are very minimal when the prior varies. When the clusters are temporally constant (Case 1) **ST-A** and **ST-A*** generally produce lower RMSE values and higher ARI values (very close to one) than model **ST-B** and **ST-B***, excepting the scenario when $Z = 0.5$ and Inverse-Gamma$(0.001, 0.001)$ is used. When the clusters evolve over time (Case 2) **ST-B** and **ST-B*** perform better than **ST-A** and **ST-A***. When there are no clusters in disease risk ($Z = 0$), model **ST-A** and **ST-A*** produce lower RMSE values than **ST-B** and **ST-B*** regardless of the choice of the prior and **ST-A** is the best of the four in terms of cluster identification, with a median ARI of 1. In addition, estimating (ρ_s, ρ_{s_t}) (**ST-A***, **ST-B***) rather than fixing them at 0.99 (**ST-A**, **ST-B**) produces better results overall in terms of both risk estimation and cluster identification in almost all scenarios. These conclusions are consistent with those provided in the simulation study in the main paper. Therefore, our methodology appears to be robust to the choice of the hyperparameters of the prior Inverse-Gamma distribution for τ_t^2.

Table S1: Median values of the RMSE, 95% credible interval coverages of the risk estimates and adjusted Rand Index (ARI) for each model and scenario.

Performance metric	Cluster case	Z	Inverse-Gamma (IG) prior	Model			
				**ST-A**	**ST-A***	**ST-B**	**ST-B***
RMSE	**Case 1**	1	IG(1,0.01)	0.088	0.070	0.090	0.075
		1	IG(0.001,0.001)	0.088	0.067	0.090	0.074
		1	IG(0.5,0.0005)	0.088	0.066	0.090	0.072
		0.5	IG(1,0.01)	0.074	0.059	0.098	0.095
		0.5	IG(0.001,0.001)	0.100	0.057	0.099	0.095
		0.5	IG(0.5,0.0005)	0.073	0.056	0.099	0.095
	**Case 2**	1	IG(1,0.01)	0.132	0.128	0.090	0.076
		1	IG(0.001,0.001)	0.132	0.126	0.091	0.075
		1	IG(0.5,0.0005)	0.132	0.127	0.090	0.076
		0.5	IG(1,0.01)	0.115	0.111	0.102	0.101
		0.5	IG(0.001,0.001)	0.115	0.111	0.099	0.102
		0.5	IG(0.5,0.0005)	0.115	0.110	0.102	0.101
	- -	0	IG(1,0.01)	0.024	0.034	0.082	0.071
		0	IG(0.001,0.001)	0.023	0.034	0.070	0.071
		0	IG(0.5,0.0005)	0.023	0.033	0.069	0.072
Coverage probability	**Case 1**	1	IG(1,0.01)	0.975	0.973	0.968	0.954
		1	IG(0.001,0.001)	0.976	0.958	0.970	0.936
		1	IG(0.5,0.0005)	0.976	0.942	0.969	0.919
		0.5	IG(1,0.01)	0.968	0.974	0.927	0.922
		0.5	IG(0.001,0.001)	0.939	0.958	0.927	0.893
		0.5	IG(0.5,0.0005)	0.969	0.946	0.926	0.876
	**Case 2**	1	IG(1,0.01)	0.932	0.942	0.964	0.969
		1	IG(0.001,0.001)	0.933	0.942	0.963	0.952
		1	IG(0.5,0.0005)	0.934	0.942	0.964	0.934
		0.5	IG(1,0.01)	0.930	0.928	0.901	0.887
		0.5	IG(0.001,0.001)	0.930	0.930	0.904	0.845
		0.5	IG(0.5,0.0005)	0.931	0.931	0.899	0.817
	- -	0	IG(1,0.01)	0.989	0.994	0.703	0.911
		0	IG(0.001,0.001)	0.961	0.978	0.720	0.843
		0	IG(0.5,0.0005)	0.925	0.955	0.698	0.803
Adjusted Rand Index (ARI)	**Case 1**	1	IG(1,0.01)	1	1	0.986	0.976
		1	IG(0.001,0.001)	1	1	0.986	0.976
		1	IG(0.5,0.0005)	1	1	0.985	0.975
		0.5	IG(1,0.01)	0.995	1	0.851	0.846
		0.5	IG(0.001,0.001)	0.541	1	0.855	0.841
		0.5	IG(0.5,0.0005)	0.994	1	0.847	0.846
	**Case 2**	1	IG(1,0.01)	0.367	0.386	0.987	0.987
		1	IG(0.001,0.001)	0.347	0.384	0.987	0.987
		1	IG(0.5,0.0005)	0.367	0.384	0.987	0.987
		0.5	IG(1,0.01)	0	0.390	0.671	0.707
		0.5	IG(0.001,0.001)	0	0.388	0.733	0.687
		0.5	IG(0.5,0.0005)	0	0.389	0.628	0.667
	- -	0	IG(1,0.01)	1	0	0	0
		0	IG(0.001,0.001)	1	0	0	0
		0	IG(0.5,0.0005)	1	0	0	0

4 Summary of model performance under the model-free scenarios

In this section we assess the performance of the proposed clustering models under a set of model-free scenarios. In these scenarios disease risks for areas with high, medium and low risk levels are respectively fixed at $\{\exp(Z), 1, \exp(-Z)\}$, rather than being generated by simulating spatial random effects using a multivariate Gaussian distribution with a Leroux CAR covariance structure and a piecewise constant mean as described in Section 4 of the main paper. For this additional study the expected counts $\{E_{it}\}$ are taken from the motivating study (SF $= 1$), and the observed disease counts $\{Y_{it}\}$ are then generated from a Poisson distribution with mean $E_{it}R_{it}$. One hundred simulated data sets are generated for each value of $Z = 1, 0.5$ under each of Cases 1 and 2. The five models **ST-A**, **ST-B**, **ST-A***, **ST-B*** and **ST-N** are applied to each data set, and the results are displayed in Table S2. The performance of each model under the model-free scenarios is similar to that displayed in the simulation study in the main paper. **ST-N** performs poorly in terms of risk estimation compared to the clustering models in the presence of clusters. **ST-A** and **ST-A*** have lower RMSE and higher ARI values than model **ST-B** and **ST-B*** when the simulated clusters are constant over time, but **ST-B** and **ST-B*** perform better when the clusters evolve over time. Additionally, estimating (ρ_s, ρ_{s_t}) (**ST-A***, **ST-B***) rather than fixing them at 0.99 (**ST-A**, **ST-B**) produces more accurate risk estimates and cluster structures in almost all scenarios.

Table S2: Median values of the RMSE, 95% credible interval coverages of the risk estimates and adjusted Rand Index (ARI) for each model and model-free scenario.

Performance metric	Cluster case	Z	Model				
			**ST-A**	**ST-A***	**ST-B**	**ST-B***	**ST-N**
RMSE	**Case 1**	1	0.088	0.065	0.091	0.072	1.202
		0.5	0.068	0.057	0.097	0.092	0.118
	**Case 2**	1	0.132	0.129	0.088	0.070	0.887
		0.5	0.115	0.111	0.097	0.097	0.113
Coverage probability	**Case 1**	1	0.981	0.980	0.971	0.962	0.832
		0.5	0.969	0.980	0.931	0.934	0.949
	**Case 2**	1	0.933	0.942	0.964	0.975	0.809
		0.5	0.934	0.928	0.909	0.906	0.949
Adjusted Rand Index (ARI)	**Case 1**	1	1	1	0.988	0.975	- -
		0.5	0.982	1	0.857	0.868	- -
	**Case 2**	1	0.359	0.402	0.987	0.987	- -
		0.5	0	0.412	0.775	0.776	- -

5 Computational time required to fit each model

Table S3 displays the time taken to fit each of the five models to the motivating Greater Glasgow and Clyde Health Board respiratory disease data. The run times relate to a single Markov chain containing 100 000 samples with a burn-in period of 80 000, which is then thinned by 10. All models are run on an HP computer with an Intel Core i7-7700 CPU 3.60 GHz processor and 16GB of RAM. The table shows that model **ST-N** is the fastest of the five models, which is because it doesn't estimate the neighbourhood matrix within the model as the other models do. However, the clustering models only have to be fitted once to the data to estimate the cluster structure. In contrast, if model **ST-N** was fitted separately with each candidate cluster structure generated in stage one of our approach, and then the best structure was chosen via a model comparison metric, then it would have to be fitted around 70 times. Thus using model **ST-N** in this fashion would be much computationally slower than using any of the cluster models proposed here. When comparing the speed of the clustering models the table shows that models **ST-B** and **ST-B*** are slower than **ST-A** and **ST-A***, which is because they need to estimate a separate neighbourhood matrix for each time period. Additionally, models

ST-A* and **ST-B*** that estimate the spatial dependence parameters (ρ_s, ρ_{s_t}) from the data are naturally slower than models **ST-A** and **ST-B** that treat these parameters as fixed.

Table S3: Comparison of the computational time required to apply each model to the motivating data.

Model	Inference	Elapsed Time
**ST-A**	MCMC (with C++)	826.31s
**ST-A***	MCMC (with C++)	866.00s
**ST-B**	MCMC (with C++)	1 358.05s
**ST-B***	MCMC (with C++)	2 345.14s
**ST-N**	MCMC (with C++)	169.52s

6 Temporal evolution in the spatial risk surfaces

The estimated spatio-temporal variation in disease risk is displayed in Figures S2 and S3 for models **ST-A*** and **ST-B*** respectively, where the former assumes a constant cluster structure over time while the latter allows it to vary from year to year. As models **ST-A*** and **ST-B*** were shown in the main paper to represent the data better than models **ST-A** and **ST-B** in terms of DIC, the results for the former are shown here.

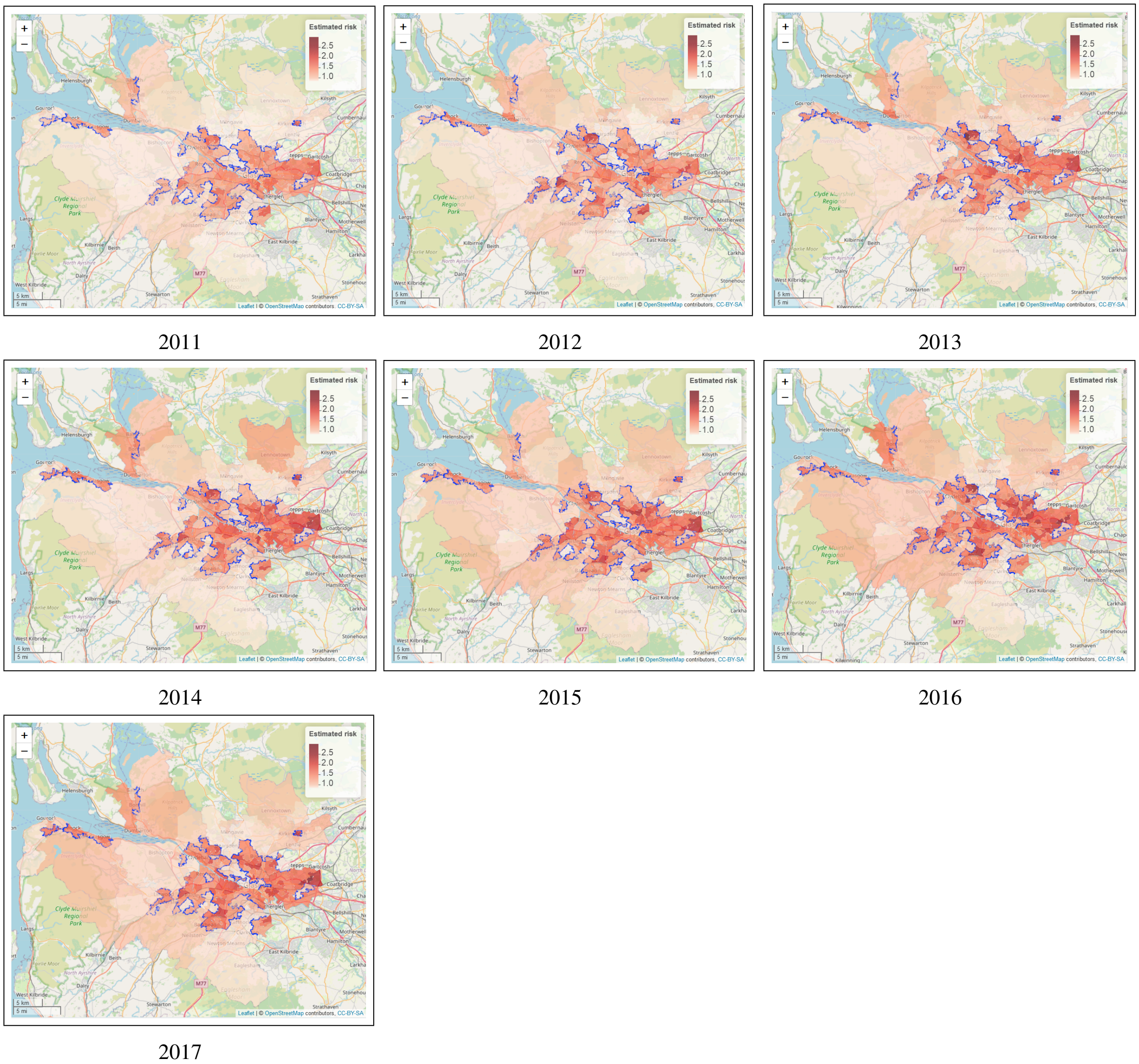

Figure S2: Maps of the estimated disease risks (posterior median) in Greater Glasgow over 2011-2017 from model **ST-A***. The estimated clusters (discontinuities), which are determined using the posterior mode of $\widetilde{\boldsymbol{W}}$, remain fixed over time and are highlighted using dots.

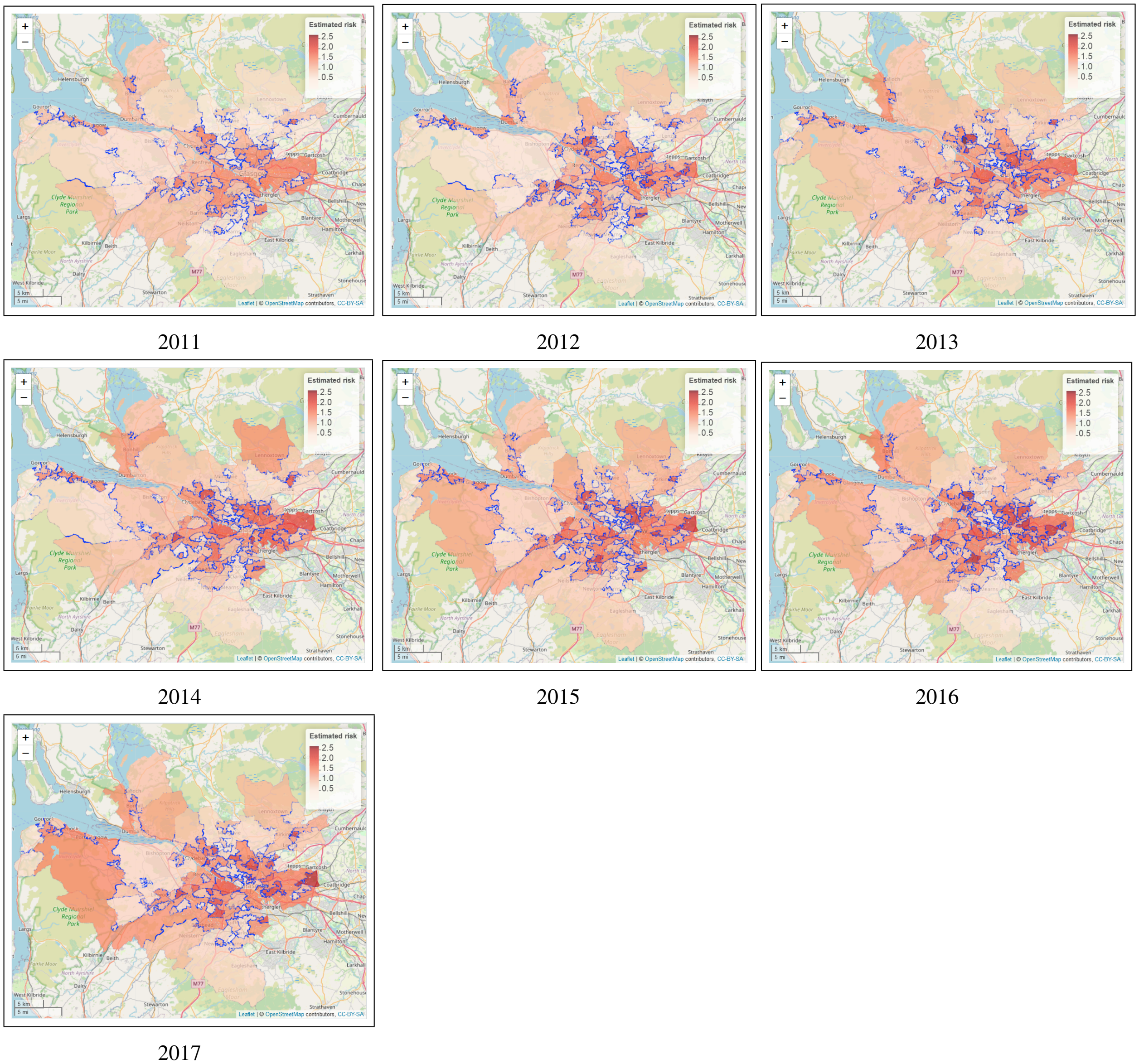

Figure S3: Maps of the estimated disease risks (posterior median) in Greater Glasgow over 2011-2017 from model **ST-B***. The estimated clusters (discontinuities), which are determined using the posterior mode of $\widetilde{\boldsymbol{W}}_t$, evolve over time and are highlighted using dots.

7 Summary of the posterior distribution of $\widetilde{\boldsymbol{W}}_t$ for model ST-B*

Figure S4 summarises the posterior distribution of $\widetilde{\boldsymbol{W}}_t$ for **ST-B*** in the motivating study for 2011, 2014 and 2017, the first, middle and last years of the study period. The figure shows that the no cluster structure (i.e., $k = 1$) is not supported by the data, with a posterior probability of zero for each time period. It also shows that the posterior distribution is mainly centered on the candidate cluster structures with the number of cluster levels (risk levels) varying between 4 and 6 clusters depending on the year.

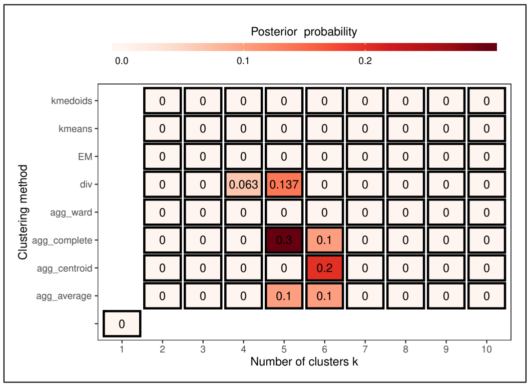

2011

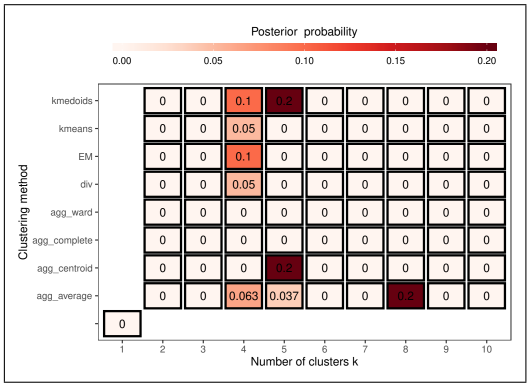

2014

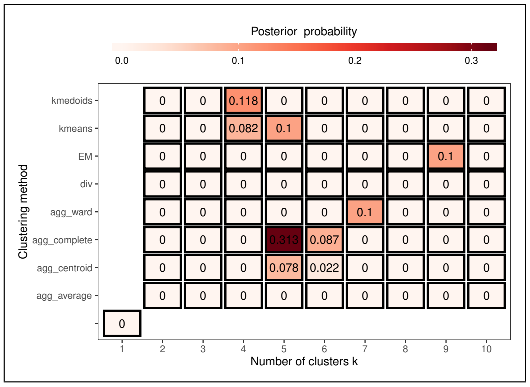

2017

Figure S4: Summary of the posterior distribution of $\widetilde{\boldsymbol{W}}_t$ over 10 Markov chains for 2011, 2014 and 2017 for model **ST-B***.
